# Terminal Respiratory Oxidases: A Targetables Vulnerability of Mycobacterial Bioenergetics?

**DOI:** 10.3389/fcimb.2020.589318

**Published:** 2020-11-23

**Authors:** Sapna Bajeli, Navin Baid, Manjot Kaur, Ganesh P. Pawar, Vinod D. Chaudhari, Ashwani Kumar

**Affiliations:** ^1^ Molecular Mycobacteriology, Council of Scientific and Industrial Research, Institute of Microbial Technology, Chandigarh, India; ^2^ Division of Medicinal Chemistry, Council of Scientific and Industrial Research, Institute of Microbial Technology, Chandigarh, India

**Keywords:** *Mycobacterium*, oxidative phoshorylation, electron transport chain, *bc1-aa3* supercomplex, cytochrome bd oxidase, Q203, respiratory inhibitors, Aurachin D

## Abstract

Recently, ATP synthase inhibitor Bedaquiline was approved for the treatment of multi-drug resistant tuberculosis emphasizing the importance of oxidative phosphorylation for the survival of mycobacteria. ATP synthesis is primarily dependent on the generation of proton motive force through the electron transport chain in mycobacteria. The mycobacterial electron transport chain utilizes two terminal oxidases for the reduction of oxygen, namely the *bc_1_-aa_3_* supercomplex and the cytochrome *bd* oxidase. The *bc_1_-aa_3_* supercomplex is an energy-efficient terminal oxidase that pumps out four vectoral protons, besides consuming four scalar protons during the transfer of electrons from menaquinone to molecular oxygen. In the past few years, several inhibitors of *bc_1_-aa_3_* supercomplex have been developed, out of which, Q203 belonging to the class of imidazopyridine, has moved to clinical trials. Recently, the crystal structure of the mycobacterial cytochrome *bc_1_-aa_3_* supercomplex was solved, providing details of the route of transfer of electrons from menaquinone to molecular oxygen. Besides providing insights into the molecular functioning, crystal structure is aiding in the targeted drug development. On the other hand, the second respiratory terminal oxidase of the mycobacterial respiratory chain, cytochrome *bd* oxidase, does not pump out the vectoral protons and is energetically less efficient. However, it can detoxify the reactive oxygen species and facilitate mycobacterial survival during a multitude of stresses. Quinolone derivatives (CK-2-63) and quinone derivative (Aurachin D) inhibit cytochrome *bd* oxidase. Notably, ablation of both the two terminal oxidases simultaneously through genetic methods or pharmacological inhibition leads to the rapid death of the mycobacterial cells. Thus, terminal oxidases have emerged as important drug targets. In this review, we have described the current understanding of the functioning of these two oxidases, their physiological relevance to mycobacteria, and their inhibitors. Besides these, we also describe the alternative terminal complexes that are used by mycobacteria to maintain energized membrane during hypoxia and anaerobic conditions.

## Introduction

*Mycobacterium tuberculosis* (Mtb) causes tuberculosis (TB) and remains one of the leading causes of human deaths worldwide from a single infectious agent (W.H. Organisation, 2019). Management of TB relies on the WHO recommended chemotherapeutic regimen known as directly observed therapy short-course (DOTS) (W.H. Organisation, 2010). DOTS utilizes the administration of four antibiotics for 6 months. Such a lengthy treatment is associated with compliance issues, and is considered as one of the reasons for the emergence of drug resistance. The number of multidrug-resistant (MDR) TB and extensively drug-resistant (XDR) TB cases are steadily rising over the years (Seung et al., 2015). Given that the antimycobacterials used in DOTS were discovered several decades back, there is an urgent need for the development of newer drugs with distinct mechanisms of action. Fortunately, recently Bedaquiline (BDQ) (W.H. Organization, 2013), Pretomanid (Keam, 2019), and Delamanid (Ryan and Lo, 2014) were approved for the treatment of MDR-TB. Of these, Pretomanid and Delamanid belong to the class of nitroimidazole. Pretomanid targets cell wall biosynthesis as well as the respiratory electron transport chain (ETC) of Mtb and thus kills both replicating and non-replicating mycobacterial cells (Manjunatha et al., 2009). Delamanid primarily inhibits mycolic acid biosynthesis in Mtb (Thakare et al., 2015). BDQ belongs to the diarylquinoline class of drugs and inhibits ATP synthesis of Mtb (Andries et al., 2005). BDQ is capable of killing both actively replicating and non-replicating persistent mycobacterial cells (Rao et al., 2008). Mtb utilizes respiratory flexibility to survive under varying environmental conditions (Trivedi et al., 2012). Due to the presence of parallel and alternative components, ETC was considered a poor drug target (Iqbal et al., 2018). However, Pretomanid and BDQ both target Mtb bioenergetics and thus have established it as a validated target. Several reviews have earlier discussed the possibility of targeting oxidative phosphorylation to develop potential therapeutic antimycobacterials (Cook et al., 2014; Bald et al., 2017; Cook et al., 2017; Iqbal et al., 2018). Two reviews were recently published emphasizing the importance of respiratory terminal oxidases in mycobacterial physiology and their potential as drug targets (Lee et al., 2020; Mascolo and Bald, 2020). In line with these reviews, here we will discuss recent studies on the contribution of respiratory terminal oxidases to mycobacterial physiology, the recent development of inhibitors targeting them, and how these could be synergistically targeted for the development of a novel regimen for the treatment of TB. Besides, we will also describe the alternative electron acceptors utilized by mycobacteria for re-oxidizing the electron carrier menaquinone for maintaining an energized membrane.

### Mycobacterial Electron Transport Chain

**Figure 1:**
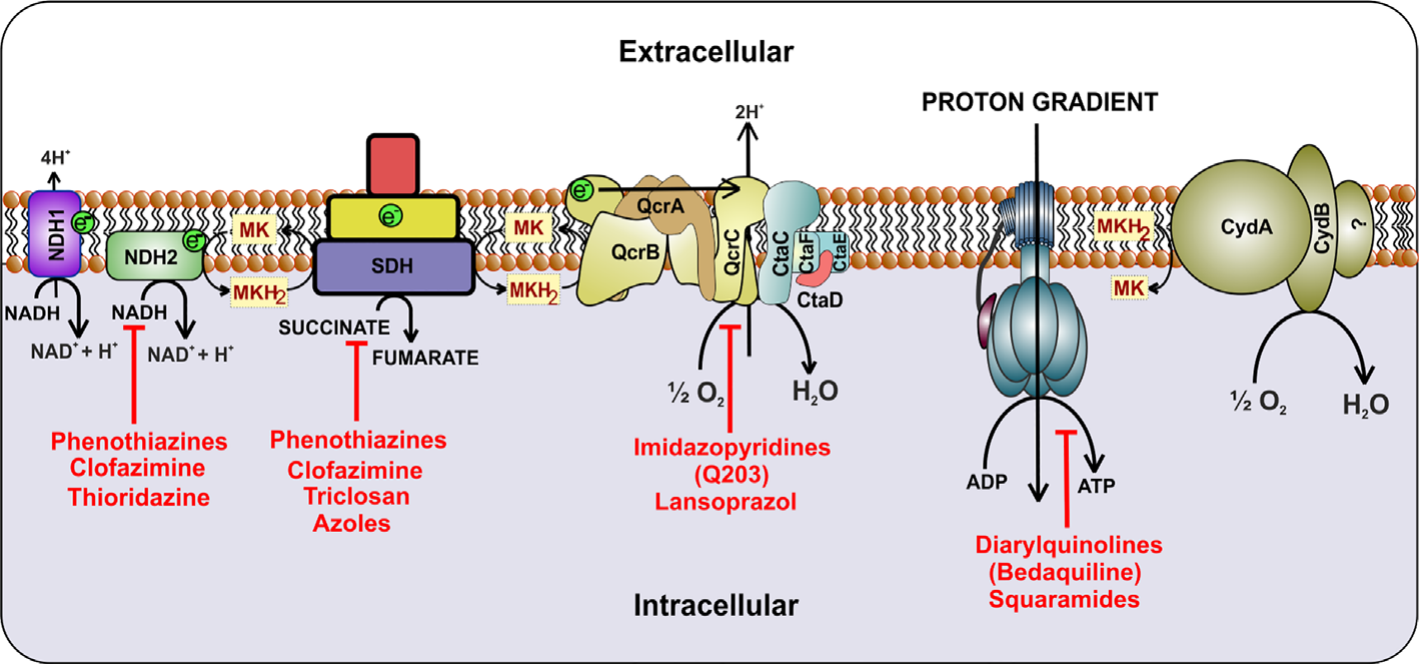
Schematic representation of the electron transport chain of mycobacteria. NADH dehydrogenase (Complex I) oxidizes NADH and transfers the electrons to cytochrome *bc_1_-aa_3_* oxidoreductase (complex III-IV) by reducing menaquinone. Alternatively, succinate dehydrogenase (complex II) uses succinate as substrate and transfers electrons to complex III *via* menaquinone. Electrons are then transferred to cytochrome *bc_1_-aa_3_* and cytochrome *bd*-type menaquinol oxidase (*bd* oxidase), which finally passes the electrons to the terminal electron acceptor, oxygen. During this process, a proton gradient is generated, which helps in the synthesis of ATP by ATP synthase. Inhibitors of the respiratory complexes are shown in red color.

ETC is utilized by microorganisms for extracting reducing power from the reduced cofactors generated during catabolic processes. It utilizes membrane-anchored dehydrogenases that accept electrons from NADH/FADH_2_ and other reduced substrates and then transfer these electrons between a series of membrane-bound multi-protein complexes, finally transferring it to the enzymes catalyzing the reduction of oxygen to water, known as terminal oxidases (Magalon and Alberge, 2016). In this process of electron transfer, protons are pumped into the periplasm, generating a proton gradient that manifests a proton motive force (PMF) (Kashket, 1985; Cook et al., 2009). This force is utilized for ATP synthesis through ATP synthase (Walker, 2013). Mycobacterial cells utilize a large number of dehydrogenases for feeding electron into the ETC (Cook et al., 2014). However, NADH/menaquinone and succinate dehydrogenase (SDH) act as primary electron feeders in mycobacterial cells (Cook et al., 2014; Iqbal et al., 2018). Mtb contains a proton-pumping type I NADH dehydrogenase encoded by *nuoABCDEFGHIJKLMN* operon and two non-proton pumping type II NADH dehydrogenases encoded by *ndh* (Rv1854c) and *ndhA* (Rv0392c) (Cook et al., 2014; Iqbal et al., 2018). Most of the mycobacterial species contain two isoforms of SDH; SDH1 and SDH2 (Pecsi et al., 2014). In Mtb, the deletion of *sdh1* disturbs the rate of respiration and leads to the inability to survive the long-term stationary phase. During aerobic growth, SDH1 functions as SDH, while SDH2 is dispensable for this catalysis (Hartman et al., 2014). SDH couples tricarboxylic acid cycle (TCA) with the ETC and feeds electron to the menaquinone pool. The pool of reduced menaquinones is then oxidized by two distinct terminal oxidases, namely menaquinol-cytochrome *c* oxidase (also known as cytochrome *bc_1_-aa_3_* complex) and cytochrome *bd*-type menaquinol oxidase (*bd* oxidase) (Cook et al., 2014; Iqbal et al., 2018). These terminal oxidases transfer electrons from menaquinol to oxygen, and during this process, reduce oxygen to water. PMF generated through this cascade of electron transfer is utilized for the synthesis of ATP *via* ATP synthase (**Figure 1**). Here it must be noted that, unlike *Escherichia coli* and many other bacterial species that can survive using substrate-level phosphorylation on fermentable carbon source, Mtb cannot grow using substrate-level phosphorylation and is dependent upon ATP synthase for ATP synthesis and growth on fermentable and non-fermentable carbon sources (Tran and Cook, 2005).

### Terminal Respiratory Oxidases

As mentioned above, mycobacterial cells use two terminal oxidases, namely, cytochrome *bc_1_-aa_3_* supercomplex and cytochrome *bd* oxidase (Cook et al., 2014; Iqbal et al., 2018). Both of these contribute to the generation of PMF through the release of protons from menaquinol into periplasmic space. However, cytochrome *bc_1_-aa_3_* complex is energetically more efficient as it pumps additional protons into the periplasm. In Gram-negative bacteria and the mitochondria of the eucaryotic cells, complex III (cytochrome *bc_1_*) is linked to the complex IV (cytochrome *aa_3_*) by a soluble cytochrome *c*. However, in *Mycobacterium*, complex III (cytochrome *bc_1_*) is fused with the complex IV (cytochrome *aa_3_*) to make a super complex (Megehee et al., 2006; Kim et al., 2015). This arrangement precludes the requirement of a soluble cytochrome *c*. A similar respiratory supercomplex is found in several genera under the phylum *Actinobacteria* (Kao et al., 2016), including *Corynebacterium* (Niebisch and Bott, 2003) and *Rhodococcus* (Sone et al., 2003). Although cytochrome *bd* oxidase is less energy efficient than the cytochrome *bc_1_-aa_3_* complex, it is more versatile, has a higher affinity for oxygen (D’Mello et al., 1996), is induced under hypoxic stress (Parish et al., 2003), and also could help with the detoxification of hydrogen peroxide (Lindqvist et al., 2000) and antibacterials (Mascolo and Bald, 2020). Besides these main terminal oxidases, mycobacterial cells are equipped with alternative oxidases/hydrogenases that function during hypoxia or absence of oxygen. These oxidases/hydrogenases help mycobacteria in the sustenance of bioenergetics but are unable to support mycobacterial growth. Thus, these are believed to play an essential role in persistence and survival during stress conditions. In the following sections, we will try to make a case for the terminal oxidases as an important and synergistic drug target aimed at the development of novel antimycobacterials.

## Cytochrome *bc_1_-aa_3_* Complex and its Inhibitors

In the following section, we will describe the evolving understanding of the role of the cytochrome *bc_1_-aa_3_* complex in mycobacterial physiology. We will also summarize the current knowledge of the inhibitors of the mycobacterial, the cytochrome *bc_1_-aa_3_* complex.

### *bc_1_-aa_3_* Oxidoreductase Complex

In bacterial and mitochondrial respiratory chains, cytochrome *bc_1_* (or complex III) extracts electrons from ubiquinone/menaquinone and then transfers them to membrane-anchored cytochrome *c*. Cytochrome *bc_1_* pumps protons in the periplasmic space during electron transfer to cytochrome *c* (Berry et al., 2000). Cytochrome *c* then passes electrons to cytochrome *c* oxidase or cytochrome *aa_3_* complex (also known as complex IV) that uses these electrons for reducing oxygen and couples the electron flow with proton translocation across the membranes (Capaldi, 1990). In mycobacteria, the complex III consists of a 2Fe/2S iron-sulfur cluster present on the Rieske protein (QcrA), a cytochrome *b* (QcrB) containing two *b-*type heme groups (low and high potential), and a di-heme *c-*type cytochrome *c_1_*(QcrC) (Niebisch and Bott, 2001; Cook et al., 2017). The *bc_1_* complex is a membrane-bound “hub” involved in the ETC of phylogenetically diverse species (Mulkidjanian, 2007). This complex is involved in the oxidation of menaquinol/ubiquinol. The Q-cycle mechanism has been proposed for explaining the functioning of the *bc_1_* complex (Mitchell, 1976). According to the Q-cycle hypothesis, two electrons from ubiquinol/menaquinol are transferred to two different chains at the Q_o_-site of the complex. The first electron from quinol is transferred to the [2Fe-2S] cluster of the Rieske protein, that further transfers it to *c* type heme, and then later, the electron is passed on to complex IV. The second electron from quinol is passed to cytochrome *b*, harboring a low potential heme (b_L_) and a high potential heme (b_H_). These electrons are delivered to a second quinone on the Q_i_-site, and the quinone is reduced to quinol (Crofts et al., 2003). Complex IV (cytochrome *aa_3_*) contains four redox active sites, namely CuA, CuB, heme *a*, and heme *a_3_* (Scott, 1995). CuA (located on CtaC, subunit II) harvests electron from the cytochrome *c* and passes them to heme *a* (located on CtaS, subunit I), which then passes them to the dinuclear site composed of heme *a_3_* and CuB (located on CtaD). The dinuclear site activates oxygen for reduction and rapidly passes four electrons converting it in water. During this process, besides consuming four scalar protons for protonation of oxygen, four vectoral protons are pumped out (Scott, 1995). The CtaS and CtaC are the primary electron acceptors from the bc1 complex, while the *a_3_-*CuB unit is the oxygen-reducing element (Cook et al., 2014).

**Figure 2:**
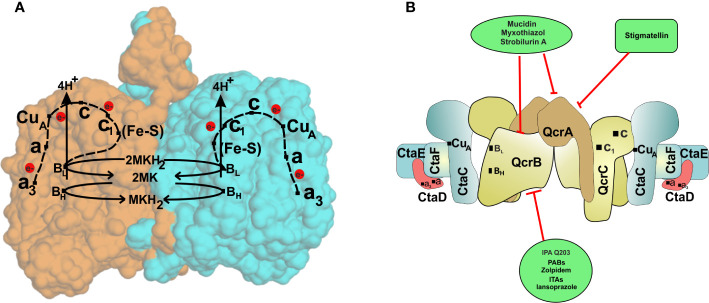
The cytochrome *bc_1_-aa_3_*oxidoreductase supercomplex. **(A)** The passage of electron through various components of the cytochrome *bc_1_-aa_3_*oxidoreductase. The crystal structure of cytochrome bc_1_-aa_3_ complex reveals its homo dimeric form. Each of the monomers is represented by a different color (orange and blue). The complex III is composed of a [2Fe-2S] cluster present on the Riske protein (QcrA), a cytochrome *b* (QcrB) containing two *b-*type heme groups (low and high potential), and a di-heme *c-*type cytochrome *c_1_*(QcrC). Electrons are initially accepted by the QcrB subunit, which is transferred to the Fe-S complex in QcrA, releasing proton in the periplasmic space. Electrons are then passed to cytochrome *c* of the QcrC subunit *via* cytochrome *c_1_*. CuA (located on CtaC, subunit II) harvests electrons from the cytochrome *c* and transfers them to heme *a* (located on CtaS, subunit I), which then passes them to the dinuclear site composed of heme *a3* and CuB (located on CtaD). The dinuclear site activates oxygen for reduction and rapid transfer of four electrons converting it to water. **(B)** Schematic representation of cytochrome *bc_1_-aa_3_*subunits and their inhibitors. Cytochrome *bc_1_-aa_3_*forms complex III and IV of the ETC of Mycobacterium species and has proved to be novel drug target against the bacteria.

In recent years, the concept of respirasomes has emerged (Krause et al., 2004). This concept suggests that different respiratory complexes associate with each other to make a supramolecular complex called “respirasome.” The organization and composition of such supercomplexes are believed to be dynamic and change with cellular energy requirements (Krause et al., 2004). In mitochondria, mostly complex I, complex III, and complex IV associate with each other in such supercomplexes (Krause et al., 2004; Lenaz and Genova, 2010; Dudkina et al., 2011). In line with these studies, a respiratory supercomplex consisting of complex III and complex IV has been reported for mycobacteria (Megehee et al., 2006; Kim et al., 2015). The mycobacterial cytochrome *bc_1_-aa_3_* supercomplex contains tightly associated menaquinol reductase and an *aa_3_* oxidase (Megehee et al., 2006; Kim et al., 2015; Gong et al., 2018). Importantly, cytochrome *c* is fused to the *bc_1_* complex in *Mycobacterium* (Megehee et al., 2006). Another important difference of mycobacterial *bc_1_-aa_3_* super complex from mitochondrial and eukaryotic III-IV supercomplex is that the former is tightly associated while the latter is only loosely associated. The atomic structure of cytochrome *bc_1_-aa_3_* complex of *M. smegmatis* was recently elucidated using cryo-electron microscopy (Gong et al., 2018; Wiseman et al., 2018). These studies revealed detailed features of supercomplex, which are specific to mycobacteria. These studies suggested that around 20 subunits associate to form the respiratory supercomplex in mycobacteria. The core of the supercomplex is composed of a *bc_1_* dimer that is sandwiched between *aa_3_* complexes on each side (Gong et al., 2018; Wiseman et al., 2018). Interestingly, QcrA of mycobacteria possesses a roof-like structure towards the periplasm that facilitates the dimerization of the *bc_1_* complex. This feature is unique to mycobacterial QcrA. The cyro-EM structure also revealed the role of previously unknown subunits, namely, Cta I, Cta J, LpqE, and prokaryotic respiratory supercomplex association factor 1 (PRSAF1) (Gong et al., 2018; Wiseman et al., 2018). LpqE and PRSAF1 are present at the interface of the *bc_1_*complex and *aa_3_* complex. The structural analysis also revealed an association of superoxide dismutase (SOD) with the *bc_1_-aa_3_* complex supercomplex (**Figure 2A**). These studies suggested that SOD may play a role in the detoxification of ROS formed by the *bc_1_*complex.

Importantly, since the *bc_1_-aa_3_* complex supercomplex is critical for the optimal growth of mycobacterial cells, the expression of its subunits is tightly controlled. Under conditions favoring aerobic respiration, the *bc_1_*-*aa_3_* supercomplex is the primary respiratory route in Mtb, yielding more ATP, and thus is essential for the mycobacterial growth (Matsoso et al., 2005; Cook et al., 2014). In response to the optimal oxygen levels, the expression of the two oxidases is regulated by mycobacteria to maximize the utilization of the terminal electron acceptor. It is assumed that the oxygen affinity of two-terminal oxidases is different (Cook et al., 2014), but whether the same is true is not analyzed. Notably, the presence of cytochrome *bc_1_-aa_3_* supercomplex in *M. smegmatis* *bd* mutant enables it to grow at a similar rate to the wild type, suggesting that *bc_1_-aa_3_* supercomplex alone can fulfill the energy needs of mycobacteria under normoxic conditions (Kana et al., 2001; Megehee and Lundrigan, 2007; Lu et al., 2015). These findings are in agreement with the observation that mutants of *M. smegmatis* and Mtb lacking QcrCAB are attenuated for growth (Kana et al., 2001; Matsoso et al., 2005; Beites et al., 2019). However, during the early phase of mice infection, the expression of the *bc_1_*-*aa_3_* complex is downregulated and stabilizes by around three weeks (Shi et al., 2005a). These observations are supported by the delayed growth of Mtb *qcrCAB* mutant in mice (Beites et al., 2019). Although a mutant of Mtb lacking a functional *bc_1_*-*aa_3_* complex can be generated, suggesting that this terminal oxidase in not essential in Mtb (Small et al., 2013; Beites et al., 2019), but several inhibitors of this complex can inhibit the Mtb growth in cultures, in macrophages, and during the infection emphasizing on its significance for mycobacterial growth.

### *bc_1_-aa_3_* Oxidoreductase Inhibitors

The role of the *bc_1_*-*aa_3_* supercomplex in optimal growth and its distinct structure and function from the mammalian respiratory system make it a relevant druggable target. Various well-characterized inhibitors of the mitochondrial, as well as mycobacterial *bc_1_-aa_3_* complex, are known. Crystallographic studies and deducing their mechanism of action has led to the development of various other inhibitors for therapeutic purposes. Usually, these inhibitors target the catalytic domains of complex utilizing the structure analogous to quinone or quinol (Abrahams et al., 2012). A variety of chemical compounds and antibiotics are known to inhibit the *bc_1_-aa_3_* complex for a long time. Myxothiazol from *Myxococcus fulvus*, a well-known antibiotic targeting the mitochondrial cytochrome *b*, was first characterized in 1984. It was shown to interact with both cytochrome *b* as well as the iron-sulfur protein of the complex resulting in the displacement of a quinone from the high-affinity binding site of the iron-sulfur protein (von Jagow et al., 1984). Antifungal antibiotics, like mucidin (from basidiomycetes *Oudemansiella mucida*) and strobilurin A (from *Strobilurins tenacellus*), also inhibit the complex by binding at the same site as that of myxothiazol (Von Jagow et al., 1986). However, another antibiotic, antimycin binds to a different location, inhibiting the oxidation of the cytochrome *b* subunit (Kucera et al., 1988). Stigmatellin, an antibiotic synthesized by *Stigmatella aurentica*, contains a 5,7-dimethoxy-8-hydroxychromone aromatic headgroup with a hydrophobic alkenyl chain in position 2. It directly binds to the cytochrome *b* Q_0_ site, associating with QcrA (von Jagow and Link, 1986).

To target a pathogen, it is important to identify compounds that are specific to the bacterial respiration pathway. This is achieved mainly by using high throughput screening (HTS), using which a plethora of cytochrome *bc_1_-aa_3_* inhibitors have been discovered until now (**Table 1**). One such screening identified the imidazo[1,2-a]pyridine (IP) series of compounds. IPs were discovered as potent inhibitors of Mtb and *M. bovis* BCG. Four IP inhibitors were specifically targeting QcrB, forcing the bacterium to use energetically less efficient cytochrome *bd* oxidase. Among these compounds, 2,6-Dimethyl-*N*-(4-(trifluoromethyl)benzyl)imidazo[1,2-*a*]pyridine-3-carboxamide with a minimum inhibitory concentration (MIC) of 0.03 µM was found to be the most potent molecule (Abrahams et al., 2012). In 2013, Pethe *et* *al.* reported a more active and less toxic class of imidazopyridine amides (IPA), targeting the Mtb cytochrome *bc_1_*. The compound, Q203 (Telacebac), was found to be active against Mtb H37Rv at an MIC_50_ of 2.7 nM *in vitro* and MIC_50_ of 0.28 nM inside the macrophages (Pethe et al., 2013). However, it must be noted that Q203 is bacteriostatic only, as mycobacteria can utilize cytochrome bd oxidase for survival in presence of Q203 (Kalia et al., 2017a). In agreement with these observations, Q203 is bactericidal for Mtb strains lacking cytochrome bd oxidase (Kalia et al., 2017a). This compound was further optimized against MDR and XDR clinical isolates in the nanomolar range (Kang et al., 2014). This leading drug candidate has recently progressed to the clinical development phase II under U.S. FDA investigational new drug application. A screen for the spontaneous resistant mutants identified the cytochrome *bc_1_* complex (*qcrB*) as the target of Q203. A mutation of Thr313 to either alanine or isoleucine was specifically involved in the resistance to Q203 (Pethe et al., 2013). In a cytochrome *bd* oxidase knockout mutant, Q203 completely inhibited the respiration (Lamprecht et al., 2016). Also, the chemical inhibition of cytochrome *bd* oxidase by aurachin D has been shown to turn the bacteriostatic activity of Q203 into bactericidal activity in Mtb and *M. smegmatis* (Lu et al., 2018). It is already evident that efflux pumps play a significant role in drug resistance in Mtb (da Silva et al., 2011). Verapamil (efflux pump inhibitor), in combination with Q203, increases the potency of the drug, showing the importance of efflux pumps mediated resistance and combination therapy with inhibitors to combat the problem (Jang et al., 2017). Putative 3D structures of the wild-type and T313A mutant along with the docking analysis with Q203 have been published, which can give insights for the resistance mechanism and will be beneficial for making better compounds within the IPA series (Ko and Choi, 2016). Apart from that, Q203 has also been evaluated against non-tuberculous mycobacteria (NTM) *M. abscessus* (Mabs), which is also quickly emerging as a health concern worldwide. It was shown that Q203 is ineffective against Mabs due to a genetic polymorphism in the target gene (QcrB). However, the complementation of Mabs *ΔqcrCAB* with chimeric Mabs *qcrCAB* (amino acids changed to Mtb *qcrCAB)* led to the susceptibility of Mabs towards this drug, suggesting the involvement of respiratory pathways in the intrinsic resistance mechanism towards drugs in Mabs (Abrahams et al., 2012). Recently, AWE402, another inhibitor belonging to the IPA class, was synthesized, which is structurally related to Q203 and shows good activity with MIC of 0.005 µM towards Mtb (Moraski et al., 2013; Ward et al., 2017). Later, a report of identification of an imidazo[1,2-a]pyridine-3-carboxamide bearing a variety of different linkers revealed 2,6-Dimethyl-*N*-[2-(phenylamino)ethyl]imidazo[1,2-a]pyridine-3-carboxamide as a potent anti-TB compound with MIC of 0.041 µM - 2.64 µM against both drug-sensitive as well as drug-resistant strains of Mtb (Lv et al., 2017). A compound belonging to this class, *N*-(4-(4-Chlorophenoxy)benzyl)-2,7-dimethylimidazo[1,2-a]pyridine-3-carboxamide (ND-09759) was also identified as a potent anti-TB molecule with an MIC of ≤0.006 µM or 0.0024 µg/ml. Its *in vivo* bactericidal activity in a mice infection model, and good pharmacokinetic (PK) properties established it as a potent anti-TB drug (Cheng et al., 2014). Imidazo[1,2-a]pyridine-3-carboxamide has also been shown to be active against *M. avium* strains *in vitro* and *in vivo* in the mouse infection model, showing the importance of these compounds as drug molecule against NTM infections as well (Moraski et al., 2016a). Using a scaffold hopping strategy, a series of novel IPA derivatives bearing an *N*-(2-phenoxy)ethyl moiety were designed, synthesized, and checked for *in vitro* inhibitory activity against both drug-sensitive H37Rv and drug-resistant clinical isolates. Compound IMB-1402 (*N*-(2-(4-Bromophenoxy)ethyl)-2,6-dimethylimidazo[1,2-a]pyridine-3-carboxamide) displayed acceptable safety and PK properties with an MIC of 0.025 µg/ml against H37Rv (Wu et al., 2016). Zolpidem (*N*,*N*-dimethyl-2-(6-methyl-2-*p*-tolyl-imidazo[1,2-*a*]pyridin-3-yl)acetamide; Ambien) is one of the best known and approved drug for the treatment of insomnia. Its striking structural similarity with imidazo[1,2-*a*]pyridine-3-carboxamides led to the discovery of Zolpidem’s anti-TB activity (Moraski et al., 2015). The rational redesign of the structural moieties found in Zolpidem resulted in the series of potent anti-TB compounds and 7-methyl-*N*-(4-methylbenzyl)-2-(*p*-tolyl)imidazo[1,2-a]pyridine-3-carboxamide was found to be the potent one with MIC of 0.004 µM demonstrating the inherent *in vitro* potency, selectivity, and low toxicity of imidazo[1,2-a]pyridines (Moraski et al., 2015).

**Table 1:** Summary of cytochrome *bc_1_*-*aa_3_* supercomplex inhibitors, their chemical structures and MIC values. Number in the parentheses denotes the references.

Chemical Class	Name of the Compound	Chemical Structure	MIC
***Imidazopyridine amides (IPAs)***	2,6-Dimethyl-*N*-(4-(trifluoromethyl)benzyl)imidazo[1,2-a]pyridine-3-carboxamide (Megehee and Lundrigan, 2007)	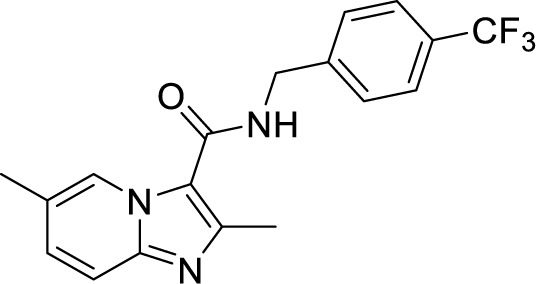	0.03 µM
	Telacebac (Q203) (Abrahams et al., 2012)	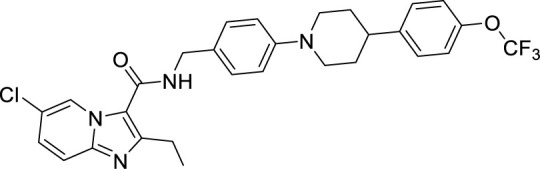	2.7 nM^a^
	AWE402 (Kang et al., 2014; Lamprecht et al., 2016)	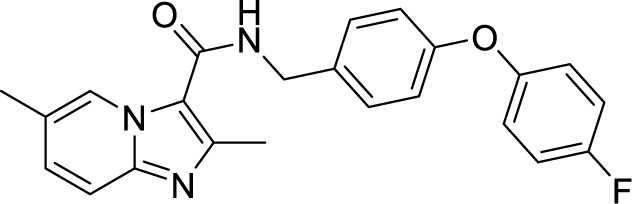	0.005 µM
	2,6-Dimethyl-*N*-[2-(phenylamino)ethyl] imidazo [1,2-a]pyridine-3-carboxamide (Lu et al., 2018)	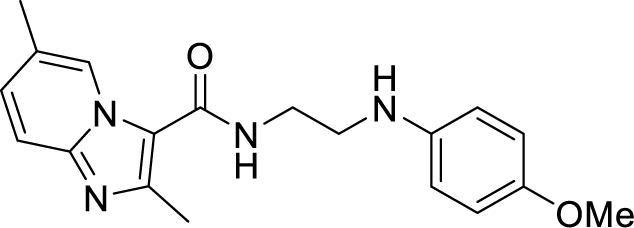	0.041 µM
	*N*-(4-(4-Chlorophenoxy)benzyl)-2,7-dimethylimidazo[1,2-a]pyridine-3-carboxamide (ND-09759) (da Silva et al., 2011)	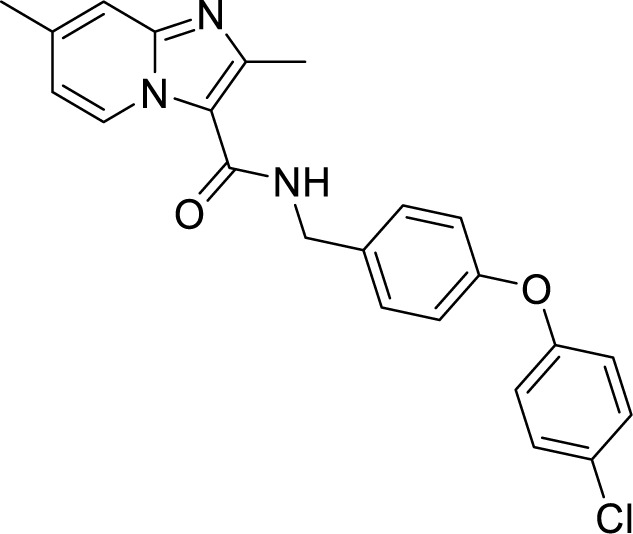	0.006 µM
	*N*-(2-(4-Bromophenoxy)ethyl)-2,6-dimethylimidazo[1,2-a]pyridine-3-carboxamide (IMB-1402) (Ko and Choi, 2016)	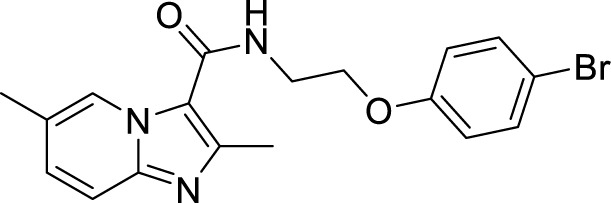	0.027 µg/ml
	Zolpidem (Ward et al., 2017)	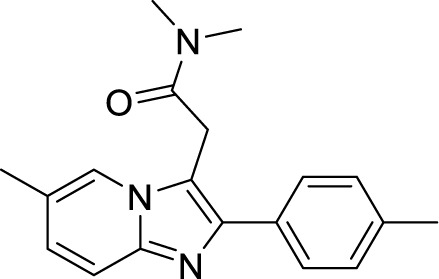	10-50 µM
	7-Methyl-*N*-(4-methylbenzyl)-2-(*p*-tolyl)imidazo[1,2-a]pyridine-3-carboxamide (Ward et al., 2017)	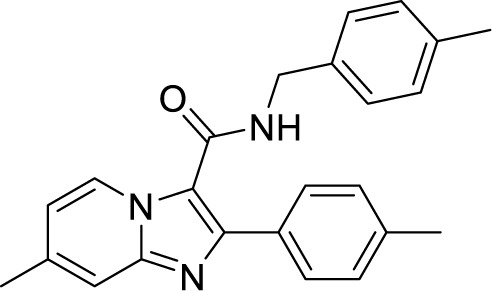	0.004 µM
***Imidazopyridine ethers (IPEs)***	4-Fluoro-*N*-(3-((2-(4-fluorophenyl)-6-methylimidazo[1,2-a]pyridin-3-yl)oxy)propyl)-3-methylaniline (Moraski et al., 2013)	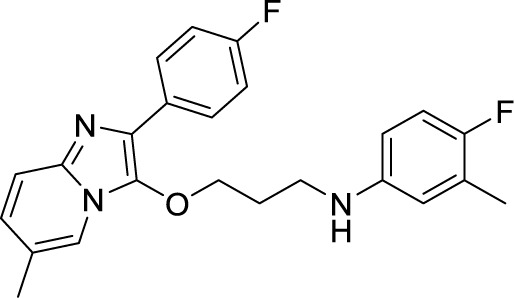	0.03 µM
***Pyrazolopyridine carboxamide (PPA)***	TB47 (Lv et al., 2017)	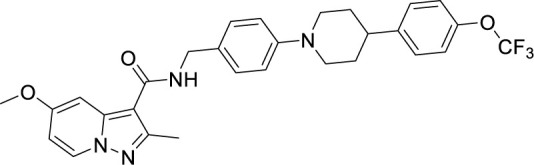	0.016 µg/ml
***Imidazo[2,1-b]thiazole-5-*** ***carboxamide***	ND-11543 (Moraski et al., 2016a)	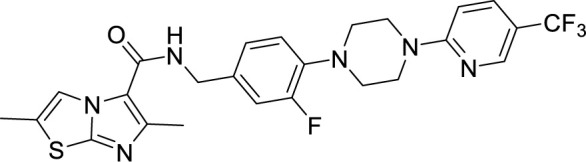	0.004 µM
***Pyrrolo[3,4-c]pyridine-*** ***1,3(2H)-dione***	7-Amino-2-(3-chlorobenzyl)-4-methyl-6-(3-methyl-1,2,4-oxadiazol-5-yl)-1*H*-pyrrolo[3,4-c]pyridine-1,3(2*H*)-dione (Wu et al., 2016)	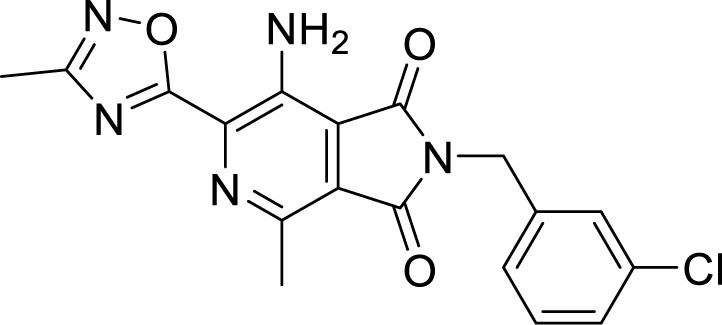	0.065 µM
***Quinazolinamine***	2-(Ethylthio)-7,8-difluoro-*N*-methylquinazolin-4-amine (Compound 11726148) (Moraski et al., 2015)	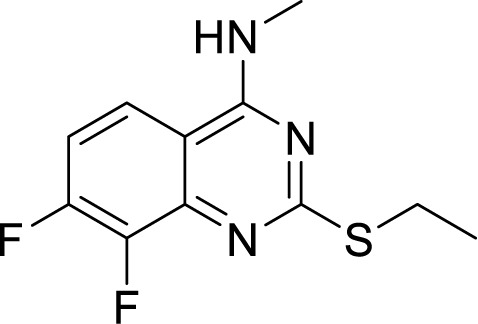	0.05 µg/ml
***Morpholinothiophene***	5-Morpholino-3-phenethoxythiophene-2-carboxamide (Tantry et al., 2017)	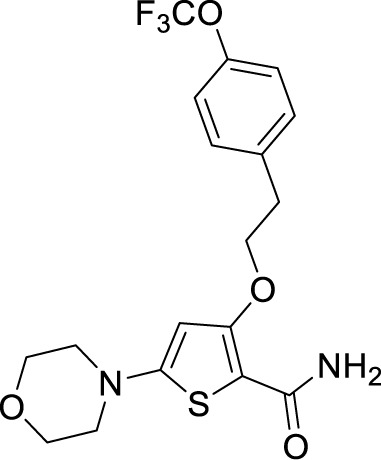	0.024 µM
***Thieno-pyrimidines***	CWHM-1023 (Lu et al., 2019)	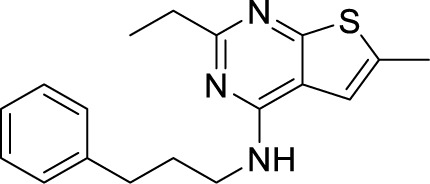	83 ± 5.4 nM^a^
***2-(Quinolin-4-yloxy)*** ***Acetamide***	GSK 358607A (Moraski et al., 2020)	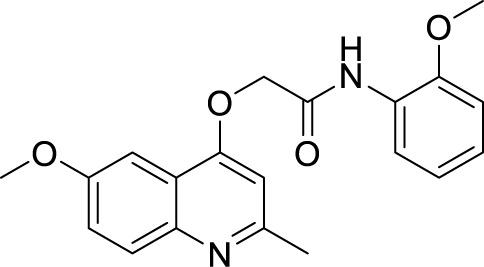	1.11 µM
	2-((6-Methoxy-2-methylquinolin-4-yl)oxy)-N-(naphthalen-2-yl)acetamide (INCT-TB422) (Cleghorn et al., 2018)	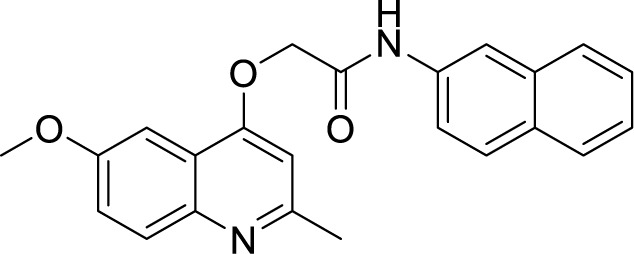	0.05 µM
	2-((6-Methoxy-2-methylquinolin-4-yl)oxy)-*N*-(4-pentylphenyl)acetamide (Cleghorn et al., 2018)	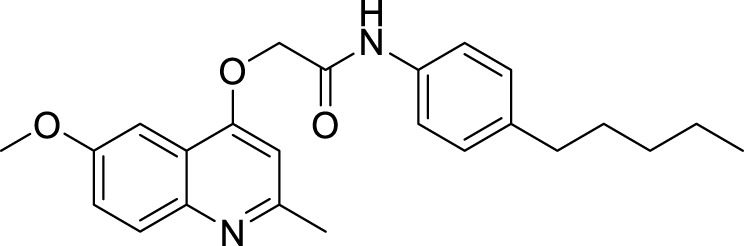	0.005 µM
***Arylvinylpiperazine amide***	AX-35 (Ballell et al., 2013)	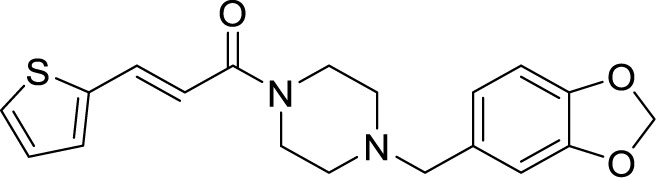	0.3 µM
***Benzimidazole***	*N*-(3-(2-Ethyl-6-methyl-1*H*-benzo[d]imidazol-1-yl)propyl)aniline (IDR-0578347) (Pitta et al., 2016)	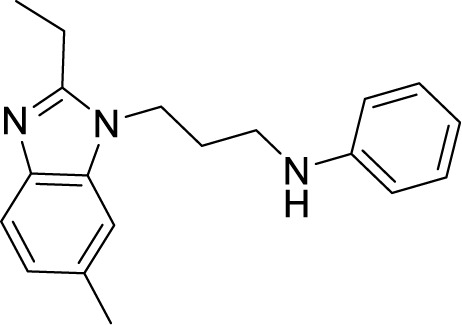	0.056 µM^b^
	Lansoprazole (Pissinate et al., 2016; Borsoi et al., 2020)	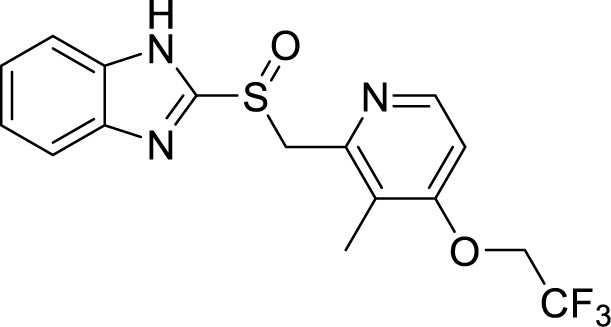	1.13 µM
***Quinolinone***	MTC420 (Foo et al., 2018)	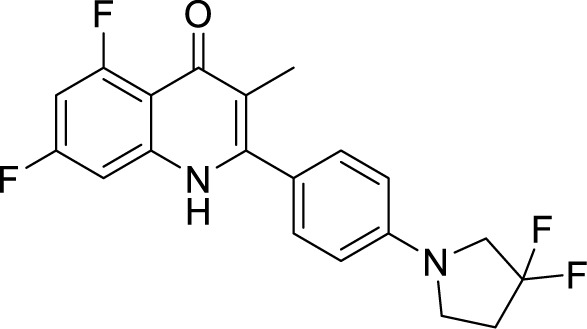	0.14 µM^c^
	SCR0911 (Ananthan et al., 2009)	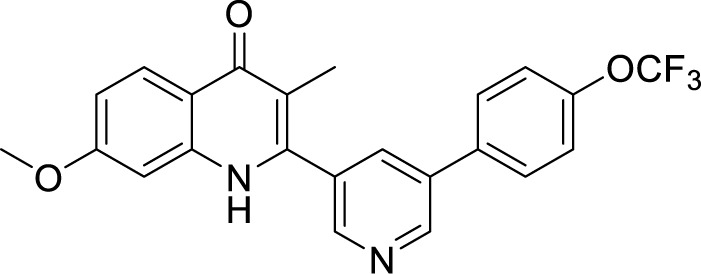	272 µM^a^

^a^MIC_50_ value. ^b^MIC_99_ value. ^c^MIC_50_ (MDR Mtb).

Another robust screening of 900,000 compounds and their SAR analysis revealed imidazo[1,2-a]pyridine ethers as inhibitors of ATP synthesis and targeting cytochrome *bc_1_*oxidase of Mtb. This screening resulted in the discovery of 4-Fluoro-*N*-(3-((2-(4-fluorophenyl)-6-methylimidazo[1,2-a]pyridin-3-yl)oxy)propyl)-3-methylaniline as anti-TB agent having an MIC of 0.03 µM against Mtb (Tantry et al., 2017).

Similarly, due to the structural similarities with Q203, TB47, a pyrazolo[1,5-a]pyridine-3-carboxamide, was identified as a potent anti-TB drug molecule, showing MIC between 0.016 and 0.500 μg/ml against various clinical strains of sensitive, MDR as well as XDR strains of Mtb. It was shown to efficiently inhibit oxygen consumption in the cytochrome *bd* mutant strain of *M. smegmatis.* The target of this compound was found to be cd2-loop of *qcrB* (H190 in *M. smegmatis*). Metabolomics profiling suggested the accumulation of TCA cycle intermediates linked to reducing-equivalents upon TB47 treatment, suggesting the toxicity by the compound to be mediated by metabolic redox stress. Like other QcrB inhibitors, it shows a bacteriostatic effect. However, upon the deletion of cytochrome *bd* oxidase, it becomes bactericidal. Also, in mice model of Mtb infection, it shows promising synergy with pyrazinamide and rifampicin, proving it to be an effective lead compound for the development of novel anti-TB chemotherapy (Lu et al., 2019).

Later, another class of compound targeting QcrB, the imidazo[2,1-*b*]thiazole-5-carboxamides were identified as a promising new scaffold, showing potent anti-TB activity against Mtb *in vitro*, inside macrophages, and MDR-TB with very low cytotoxic activity (Moraski et al., 2016b). These compounds show MIC_90_ ranging from 0.0625 µM - 2.5 µM against Mtb, along with potency towards mono-drug resistance strains in a concentration ranging from 0.0017 µM - 7 µM. A recent study demonstrated its good *in vitro* ADME properties including, protein binding, CaCo-2, human microsomal stability, and CYP450 inhibition. They also demonstrated the good efficacy of a tool compound, ND-11543, in the murine TB infection model (Moraski et al., 2020).

Another HTS of the library of small polar molecules led to the identification of compounds showing activity against Mtb. Still, the presence of ester linkage imposed a question over their metabolic instability. So the compounds were optimized to get good metabolic stability in mouse PK studies yielding more stable Pyrrolo[3,4-c]pyridine-1,3(2*H*)-dione and showing MIC_90_ in the micromolar range against Mtb. Among those, 7-Amino-2-(3-chlorobenzyl)-4-methyl-6-(3-methyl-1,2,4-oxadiazol-5-yl)-1*H*-pyrrolo[3,4-c]pyridine-1,3(2*H*)-dione had an MIC of 0.065 µM (van der Westhuyzen et al., 2015). These compounds are hyperactive against cytochrome *bd* oxidase mutants, and a point mutation (Ala317Thr) in *qcrB* results in resistance towards these compounds, strongly indicating the target to be the QcrB subunit (van der Westhuyzen et al., 2015).

Further, new potent quinazoline derivatives were synthesized and investigated for anti-TB activity. This study yielded a series of 2-ethylthio-4-methylaminoquinazoline derivatives against Mtb. They tested 76 derivatives, out of which four ((11626141, 11626142, 11626252, and 11726148) had good activity (MIC below 0.09 µg/ml). These compounds had very low cytotoxicity in human hepatocytes. Compounds 11626252 (2-(ethylthio)-8-fluoro-N-methylquinazolin-4-amine) and 11726148 (2-(ethylthio)-7,8-difluoro-*N*-methylquinazolin-4-amine) were also active in mice model of TB infection, showing 0.51 log_10_ CFU/organ upon treatment for 10 days. Surprisingly, the analysis of mutants showing resistance towards these compounds identified QcrA and QcrB as the target of these compounds. This was the first report which identified QcrA as an important druggable target. Compound 11626252 is bacteriostatic, but in a strain of Mtb lacking cytochrome *bd* oxidase (H37Rv *ΔcydAB*), it becomes bactericidal (Lupien et al., 2020). This proves that combination therapy of drug molecules targeting both the branches of Mtb aerobic respiration could be a new therapeutic regimen.

An aerobic whole-cell phenotypic screening of Eli Lilly corporate library against Mtb resulted in the identification of a cluster of a novel morpholino-thiophenes series. This screen resulted in the identification of tool compounds like 5-morpholino-3-phenethoxythiophene-2-carboxamide. These compounds had MIC_90_ in the micromolar range with no cytotoxicity and were active in an acute murine model of infection. Again, the target of these compounds was QcrB, proving it to be a crucial druggable target (Cleghorn et al., 2018).

In another study, a class of QcrB inhibitors was discovered, known as 4-amino-thieno[2,3-*d*]pyrimidines, that are chemically distinct from previously identified inhibitors, adding to the growing chemical space that can be exploited for the generation of new compounds with anti-TB activity. The most potent compound from their SAR analysis yielded CWHM-1023 having MIC_50_ of 83 ± 5.4 nM against Mtb. In combination with Q203, CWHM-1023 efficiently decreased the ATP levels in *M. smegmatis* and Mtb. Also, upon the deletion of *cydA*, bacteria become more sensitive towards this compound, proving its efficacy as an anti-TB drug molecule (Harrison et al., 2019).

In 2013, GlaxoSmithKline (GSK) made the results of an anti-mycobacterial phenotypic screening publically available. A total of 177 hits were reported, belonging to different structural classes (Ballell et al., 2013). Out of them, five 2-(quinolin-4-4-yloxy)acetamides (QOAs) exhibited potent anti-mycobacterial properties (MIC_90_ of 0.3 µM - 3.3 µM). Out of them, GSK 358607A was further investigated for its anti-TB activity using a preliminary SAR, leading to the identification of four analogs that were more potent than the original inhibitor. This class was also shown to target QcrB (Phummarin et al., 2016). The problem with these compounds was their moderate metabolic stability, due to the amide group lability (Pitta et al., 2016; Giacobbo et al., 2017). SAR studies of QOAs resulted in the discovery 2-((6-Methoxy-2-methylquinolin-4-yl)oxy)-N-(naphthalen-2-yl)acetamide (INCT-TB422), with an MIC of 0.05 μM and 2-((6-Methoxy-2-methylquinolin-4-yl)oxy)-N-(4-pentylphenyl)acetamide with an MIC of 0.005 μM against Mtb H37Rv (Pissinate et al., 2016). It was shown to be active against intracellular and drug-resistant Mtb strains as well (Giacobbo et al., 2017). Recently, another group conducted molecular simplification of these compounds yielding more stable and active even towards MDR-TB. The compounds thus generated were active in a macrophage model of TB infection (Borsoi et al., 2020).

Another study was focused on arylvinylpiperazine amides, which were also identified in the screen conducted by GSK. One of them was GW861072X (AX-35), which showed MIC_90_ of 0.3 µM against Mtb and *M. bovis* BCG. Lead optimization of this compound led to the identification of analogs with potent activity against Mtb *in vitro* and inside macrophages with mild cytotoxicity. These compounds were also active in an acute mouse model of TB infection, proving to be a good candidate against Mtb (Foo et al., 2018).

Further, HTS of a chemical diversity library containing 100,997 compounds was screened to identify a specific class of compounds showing anti-TB activity. It led to the discovery of the phenoxy-alkyl benzimidazole (PAB) class of compounds. They were showing a nanomolar range of MIC_90_ and very low cytotoxicity (Ananthan et al., 2009; Chandrasekera et al., 2015). Later, isolation of mutants resistant to PAB compounds showed mutations either in *rv1339*, a gene of unknown function, or *qcrB* (Chandrasekera et al., 2017). SAR studies of PABS yielded improved compounds with alkylbenzimidazole moiety. Several compounds were even active against intracellular bacteria, out of which the compound *N*-(3-(2-ethyl-6-methyl-1*H*-benzo[d]imidazol-1-yl)propyl)aniline (IDR-0578347) was most potent with MIC_99_ of 0.056 ± 0.020 µM (Chandrasekera et al., 2017). Already known gastric proton pump inhibitor, lansoprazole (LPZ, Prevacid), which is extensively used to treat acid-related stomach disorders, was found to possess intracellular activity against Mtb. Target identification studies revealed its target to be cytochrome *bc_1_*complex by intracellular sulfoxide reduction to lansoprazole sulfide. A single nucleotide polymorphism of leucine-176 to proline in the cytochrome *b* results in the resistance towards this drug. LPZ rapidly converted to lansoprazole sulfide (LPZS), a potent anti-mycobacterial agent, cannot bind to human H^+^K^+^ -ATPase and thus can be used as a drug molecule (Rybniker et al., 2015). But *in vivo*, LPZ does not yield a sufficient amount of LPZS to be active against Mtb, while a single intravenous dose of pure LPZS does results in higher tissue concentration, adequate to kill Mtb (Mdanda et al., 2017). LPSZ is highly specific and possesses potent activity against drug-resistant isolates of Mtb (Rybniker et al., 2015). Through modeling of mutant mycobacterial protein on the published QcrB protein, it was revealed that both L176P (mutation leading to resistance towards LPZS) and T313A (resistance towards IPA) were localized to the same site, i.e., ubiquinol oxidation (P site). But, L176P mutants remained susceptible to various IPA, and T313A mutants were susceptible to LPZS, revealing a different binding mechanism for them (Rybniker et al., 2015).

A compound of quinolinone class, MTC420, synthesized by Hong and coworkers, has been reported as the cytochrome *bc_1_* inhibitor with an MIC value of 0.14 μM (Hong et al., 2017). Another repurposing strategy led to the identification of an anti-malarial compound as an anti-TB agent, inhibiting cytochrome *bc_1_* oxidase, SCR0911. It showed an MIC_50_ of 272 ± 41 μM against *M. smegmatis* and 107 ± 5.8 μM against *M. bovis* BCG. Also, SCR0911 resulted in rapid intracellular ATP depletion in mycobacteria, indicating the interruption of electron flow. Docking studies revealed the Q_i_ site of mycobacterial cytochrome *bc_1_*to be the potential target of this drug. This opens the door for the SAR analysis of this compound for improved and specific drug molecules against Mtb (Chong et al., 2020).

Despite the high vulnerability of this respiratory branch, it is surprising to note that the screening of compounds targeting the supercomplex has majorly identified the compounds inhibiting the narrow region of QcrB subunit (Arora et al., 2014; Rybniker et al., 2015) (**Figure 2B**). A recent optimal homology model of Mtb QcrB using *M. smegmatis* QcrB as a template was built. This could provide a more in-depth insight into the interaction of Mtb QcrB with its inhibitors (Pan et al., 2019). However, exciting drug-development has been done in recent times, the absence of *bc_1_-aa_3_*supercomplex results in the upregulation of cytochrome *bd* oxidase to meet the energetics demand of Mycobacteria (Matsoso et al., 2005; Arora et al., 2014), which demands a much better understanding of the mycobacterial ETC to target the mycobacterial cytochrome *bc_1_-aa_3_* oxidoreductase supercomplex. Although the same is not valid in the case of the clinical isolate, which regulates cytochrome *bd* expression more tightly (Rybniker et al., 2015).

## Cytochrome bd Oxidase and its Inhibitors

In the following section, we will describe the current understanding of the role of cytochrome *bd* oxidase in mycobacterial physiology. We will also describe currently known inhibitors of the mycobacterial cytochrome *bd* oxidase.

### Cytochrome *bd* Oxidase

Cytochrome *bd* oxidase is confined to the prokaryotic world and thus is considered a plausible drug target. Unlike cytochrome *c* oxidase, it does not pump protons to the periplasm and therefore is not energy efficient (Borisov et al., 2011). However, it still helps in the generation of PMF through the vectorial charge transfer of protons (Borisov et al., 2011). It has been purified and characterized from *E. coli* (Miller and Gennis, 1983), *Azotobacter vinelandii* (Junemann and Wrigglesworth, 1995), *Corynebacterium glutamicum* (Kusumoto et al., 2000), and *Geobacillus thermodenitrificans* (previously *Bacillus Stearothermophilus*) (Sakamoto et al., 1996). The long-awaited crystal structure of cytochrome *bd* oxidases was recently solved for enzymes from *Geobacillus thermodenitrificans* and *E. coli* (Safarian et al., 2016; Safarian et al., 2019; Theßeling et al., 2019). Mycobacterial cytochrome *bd* oxidase is not extensively studied and is assumed to share similar functions to the closest structural bacterial homologs. Analysis of the Mtb genome suggests that Mtb cytochrome *bd* oxidase comprises of two main canonical subunits, namely CydA and CydB. In the Mtb genome, genes encoding for CydA, CydB, CydC, and CydD are present in an operon. The function of CydC and CydD is not clear, but they are assumed to be cytochrome ABC type cysteine exporter, which may facilitate the proper assembly of the cytochrome *bd* oxidase (Cruz-Ramos et al., 2004). In *M. smegmatis*, the *cydDC* and *cydAB* are located on separate operons parted by 100 base pairs (Aung et al., 2014). Sequence comparison of CydA and CydB across several mycobacteria reveal higher similarity in the sequence of *cydA* compared to *cydB*. This observation is in concordance with the norm of asymmetrical evolution of cytochrome *bd* subunits (Hao and Golding, 2006). It has been demonstrated that sensitivity to respiratory inhibitors such as hydrogen cyanide and pyocyanin significantly varies by microevolution of *cydB* (Voggu et al., 2006); however, in mycobacteria, it has not been explored yet. The sequences of *cydA* and *cydB* in Mtb is 100% identical to *M*. *bovis* BCG, making BCG a useful model organism to study cytochrome *bd* oxidase. Subunit CydA is involved in the oxidation of the menaquinol. It contains a q loop that binds to menaquinol and three heme groups that act as redox centers for transferring the electron flux across the terminal oxidase. These three heme are low spin cytochrome *b_558_*, high spin-*b_595,_* and chlorin type heme d. Recently, the homology model of cytochrome *bd* oxidase has been created (Sviriaeva et al., 2020) by utilizing the crystal structures of the enzymes from *G. thermodenitrificans* and *E. coli* (Safarian et al., 2016; Safarian et al., 2019). Comparing the mycobacterial and bacterial amino acid sequence shows that the menaquinol binding Q loop region in mycobacterial cytochrome *bd* oxidase harbors various residues that are unique and different from bacterial orthologs (Sviriaeva et al., 2020). Given the uniqueness of the additional amino acid stretches in CydAB and the role of these in the function of cytochrome *bd* oxidase, these could be utilized in discovering mycobacteria specific inhibitors. In mycobacterial cytochrome *bd* oxidase, the presence of methionine residues around the catalytic site may be the reason for reactive oxygen species (ROS) scavenging and guarding the enzyme (Harikishore et al., 2020).

**Figure 3:**
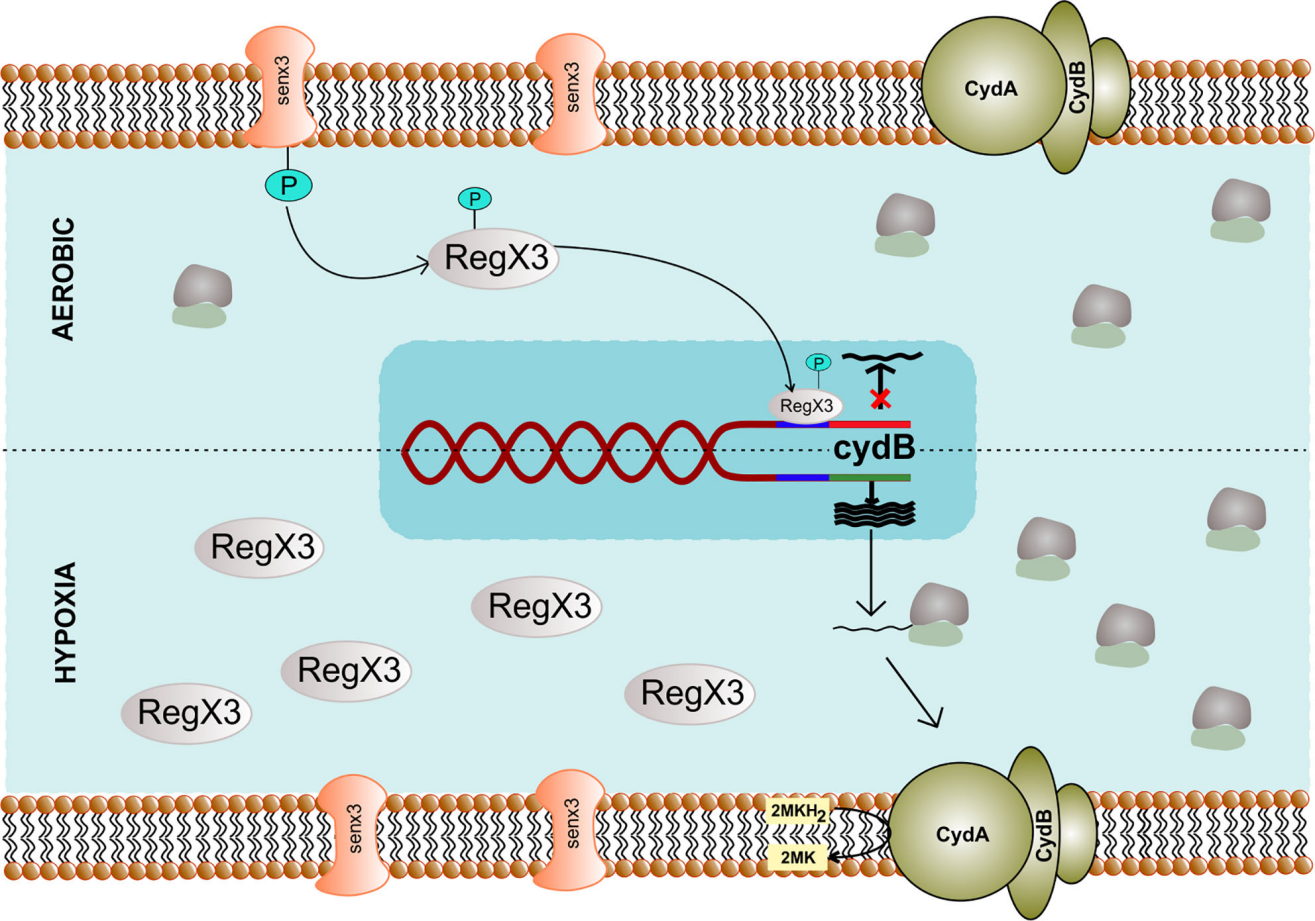
Genetic regulation of cytochrome *bd* complex. The expression of cydB component of terminal oxidase cytochrome *bd* depends upon SenX_3_ – RegX_3_ two-component system specific to *Mycobacterium.* During the aerobic condition, phosphorylated RegX_3_ binds to the promoter region of cydB and inhibits transcription. However, during hypoxia, RegX_3_ gets dephosphorylated and does not bind to the promoter of cydB, thus facilitating its expression.

A variable regulation of cytochrome *bd* expression is seen across different mycobacterial species as the *cydABDC* genes can form a polycistronic operon in various bacteria (Kabus et al., 2007). *E. coli* cytochrome *bd* possesses a higher affinity for oxygen (D’Mello et al., 1996) and is induced under hypoxic conditions (Poole and Cook, 2000). Although the oxygen affinity of the mycobacterial CydAB has not been determined yet, it is known to be induced in response to hypoxia (Kana et al., 2001; Berney and Cook, 2010). Expression of *cydAB* is regulated by ubiquinone/ubiquinol sensor ArcA/ArcB in *E. coli* (Malpica et al., 2004), while in mycobacteria, it is regulated by the oxygen sensing two-component system SenX3/RegX3 (Roberts et al., 2011; Singh and Kumar, 2015) (**Figure 3**). Additionally, cyclic AMP receptor protein regulates the expression of *cydAB* in *M. smegmatis* under hypoxic conditions (Aung et al., 2014). However, whether the same is true for the Mtb homolog of cyclic AMP receptor protein needs to be further explored. In line with these observations, the *cydAB* operon is induced in response to nitric oxide (Shi et al., 2005a) that inhibits mycobacterial respiration. Notably, the *cydAB* operon is also induced during chronic infection in mice lungs (Shi et al., 2005a), suggesting a role in mycobacterial survival in the mice lungs. Furthermore, Mtb *cydAB* mutants are attenuated for survival in mice during chronic infection (Shi et al., 2005a). These observations are in agreement with earlier observations wherein CydAB was shown to play an important role in virulence and survival of various bacterial pathogens inside the host (Juty et al., 1997; Zhang-Barber et al., 1997; Endley et al., 2001; Turner et al., 2003; Baughn and Malamy, 2004; Shi et al., 2005a; Yamamoto et al., 2005; Larsen et al., 2006). Although the precise role of CydAB in protection from the host system remains undefined, these observations suggest a role beyond maintaining cellular bioenergetics. Emerging evidence indicates that CydAB could play a role in imparting resistance towards oxidative and nitrosative stress (Junemann and Wrigglesworth, 1995; Hori et al., 1996; Winstedt et al., 1998; Lindqvist et al., 2000; Borisov et al., 2011), as well as to the low iron concentrations (Cook et al., 1998). These effects could be explained due to the capability of CydAB oxidase to detoxify ROS. *E. coli* CydAB is known to possess quinol peroxidase activity that leads to detoxification of hydrogen peroxide (Al-Attar et al., 2016). It is plausible that Mtb CydAB oxidase could also protect the bacilli from oxidative stress, but it remains to be analyzed. In agreement with this hypothesis, the cytochrome *bd* oxidase expression rises three-fold in Mtb strain lacking a functional cytochrome *bc_1_-aa_3_* supercomplex, leading to increased resistance against H_2_O_2_ (Small et al., 2013). In classical strains of *M. ulcerans* and *M. leprae*, a functional cytochrome *bd* oxidase is absent, whereas, in Mtb, the enzyme may considerably protect the bacteria from multiple environmental stresses, including low oxygen tensions and nitric oxide or peroxides during infection in the human host (Cole et al., 2001; Berney et al., 2014; Scherr et al., 2018). In *E. coli*, cytochrome *bd* oxidase is overexpressed in biofilms. Loss of this enzyme results in the impairment of biofilm formation (Beebout et al., 2019). The role of cytochrome *bd* oxidase in mycobacterial biofilm formation still remains to be established.

### Cytochrome *bd* Oxidase Inhibitors

**Figure 4:**
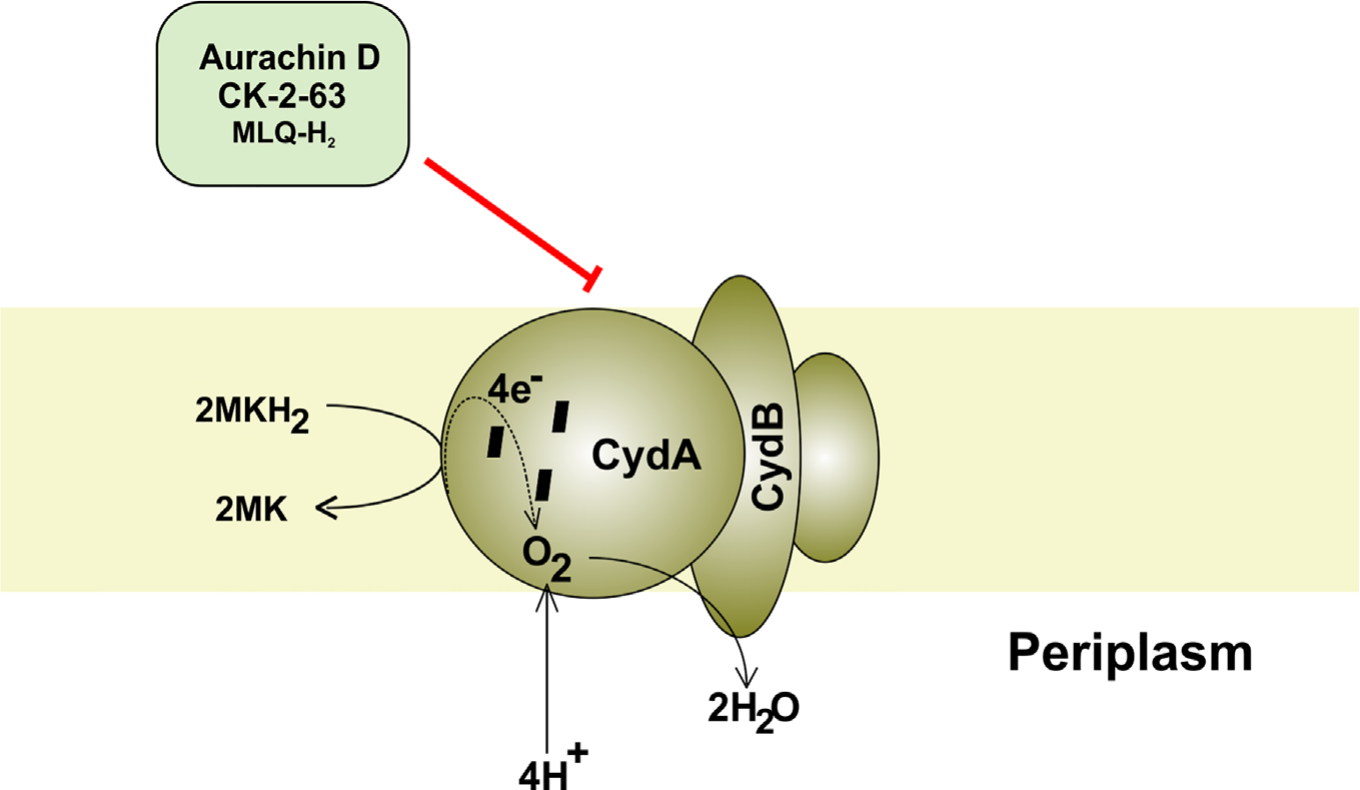
Mycobacterial cytochrome *bd* oxidase. The components and inhibitors of terminal oxidase cytochrome *bd* and the passage of electron through its various subcomponents. The electron is finally accepted by oxygen, which accepts proton from the periplasm to form water.

**Figure 5:**
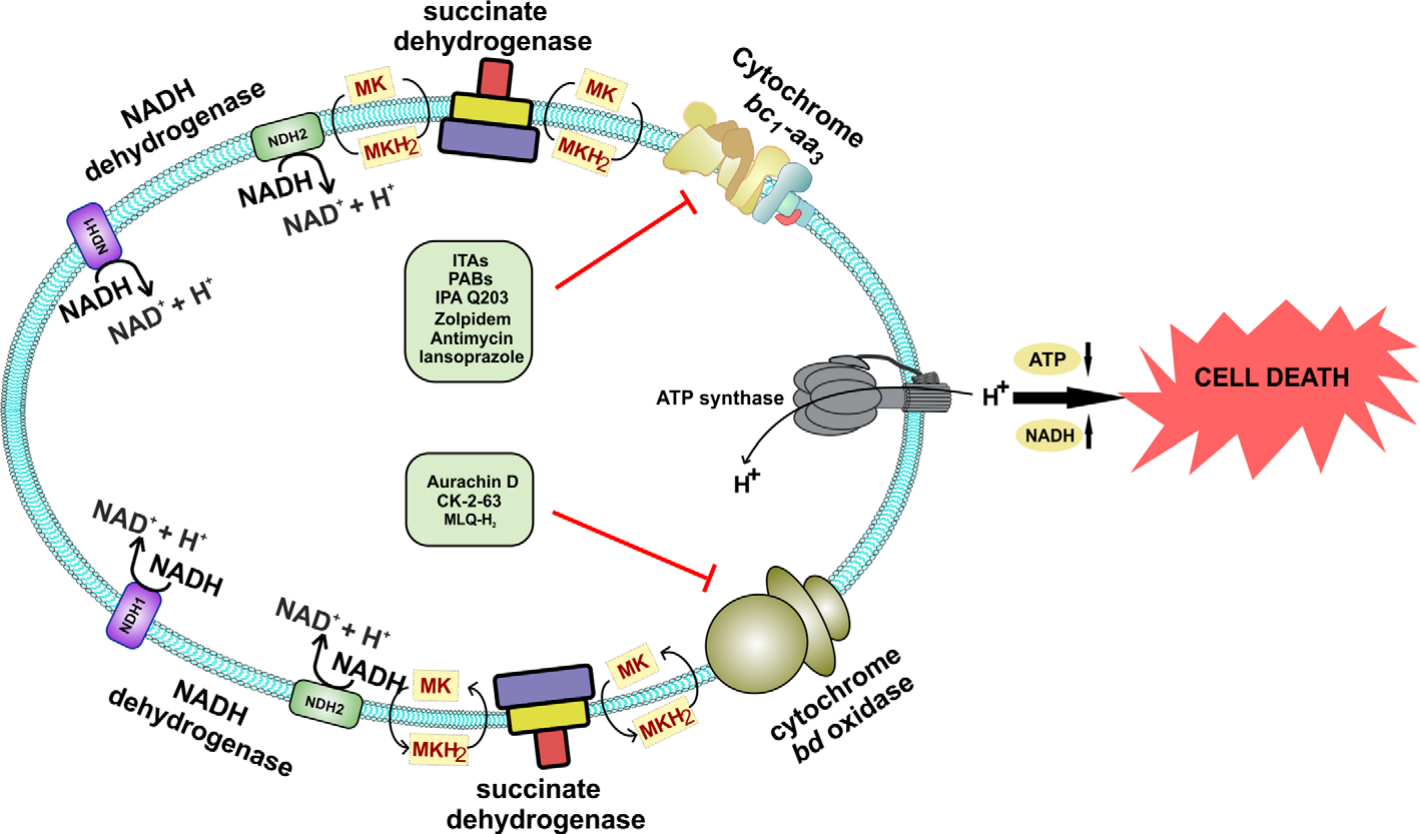
Schematic representation of the effect of inhibition of both the terminal oxidases of Mycobacterium species. Inhibition of both the terminal oxidases leads to cell death. A combination of various drugs could directly target both of these terminal oxidases, which are specific to *Mycobacterium* and could effectively kill the bacteria, thus providing a novel drug target against *Mycobacterium* species.

Cytochrome *bd* oxidase is specifically present in bacteria and thus could be used as a target for anti-TB drug development (**Figure 4**). Furthermore, mycobacterial CydAB oxidase contains an extended menaquinone binding loop, and several additional stretches of amino acids essential for its function, making it amenable to Mtb specific drug targeting (**Figure 5**). But the non-essentiality of cytochrome *bd* oxidase represents a challenge to identify its inhibitors. Not many compounds are known to inhibit cytochrome *bd* oxidase (**Table 2**). The Aurachin class of compounds is a quinone analog, which has been reported to inhibit a variety of cytochrome oxidases (Meunier et al., 1995; Debnath et al., 2012; Li et al., 2013). Aurachin D is well known among this class of compounds known to inhibit *E. coli* cytochrome *bd* oxidase (Meunier et al., 1995). It inhibits oxygen consumption in *M. smegmatis* in a dose-dependent manner. Optimized, derivatives of Aurachin D, with better ability to penetrate the mycobacterial cell wall, can become a new class of antitubercular drugs (Lu et al., 2015). Gramicidin S represents another class of compounds capable of inhibiting *bd* oxidase (Mogi et al., 2008). It is a cationic cyclic decapeptide and possesses potent antibiotic activity. Earlier models suggest that it perturbs the bacterial membrane to exert its antibiotic activity, but recent literature shows that it could also target *bd* oxidase (Mogi et al., 2008). Besides, Gramicidin S, another antimicrobial peptide Microcin J25 was shown to inhibit *E. coli* *bd* oxidase (Galvan et al., 2019). However, it remains to be seen if these antimicrobial peptides could inhibit the respiration of mycobacterial cells. Another study identified the prenylphenols class of compounds from the Kitasato Institute for Life Sciences Chemical Library to inhibit *bd* oxidases. Interestingly, prenylphenols LL-Z1272 beta and epsilon were specifically active against the *bd* oxidase (Mogi et al., 2009). Importantly, the utilization of the computational biology approach led to the identification of MQL-H2, a novel inhibitor of *bd* oxidase (Harikishore et al., 2020). This inhibitor is postulated to bind the menaquinol binding site of CydA and inhibits ATP synthesis in inverted membrane vesicles of *M. smegmatis* (Harikishore et al., 2020). In an interesting development, a quinolone derivative CK-2-63, and its analogs were identified as potent inhibitors of mycobacterial *bd* oxidase inhibitors (Ward et al., 2017). CK-2-63 alone partially inhibits the growth of Mtb *in vitro* cultures. Interestingly, this inhibitor, in combination with cytochrome *bcc* inhibitors and/or ATP synthase inhibitor Q203 was capable of killing mycobacterial cells (Ward et al., 2017).

**Table 2:** Summary of cytochrome *bd* oxidase inhibitors, their chemical structures and MIC values. Number in the parentheses denotes the references.

Compound name	Chemical structure	MIC value
**Aurachin D** (Lenaz and Genova, 2010)	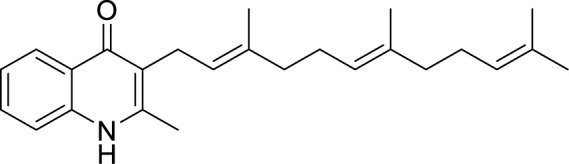	>85 µM
**MLQ-H_2_** (Berney et al., 2014)	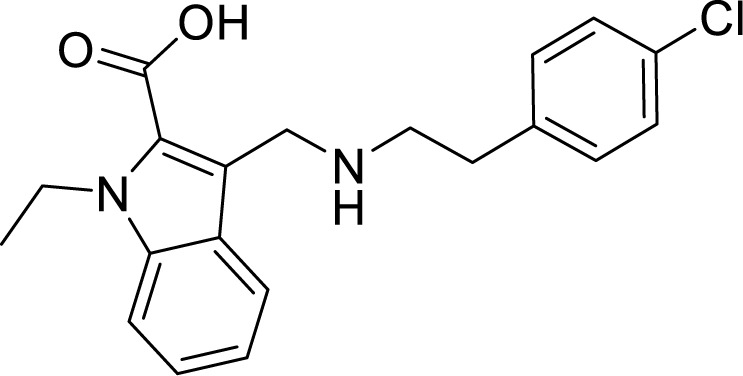	34 µM
**CK-2-63** (Kang et al., 2014)	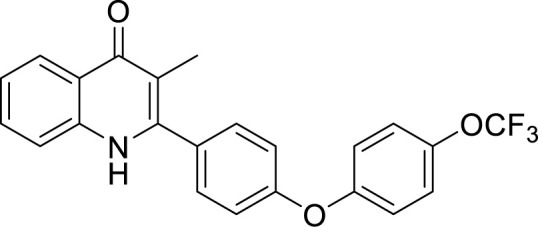	0.003 µM

## Alternative Respiratory Complexes

Respiratory complexes *bc_1_*-*aa_3_* supercomplex and cytochrome *bd* oxidase utilize oxygen as a terminal electron acceptor. However, during severe hypoxia and anoxia, oxygen is not readily available to act as an electron acceptor. Under such conditions, *E. coli* cells utilize alternative electron acceptors such as nitrate and fumarate for oxidizing the reduced pool of the electron carrier menaquinone. Importantly, under oxygen limiting conditions and upon inhibition of respiration, NADH levels are tremendously reduced along with lower levels of ATP (Watanabe et al., 2011; Bhat et al., 2016). Reoxidation of NADH is critical for mycobacterial cells to deter reductive stress and survive during such conditions (Mavi et al., 2020). Mycobacterium alters its ETC and central metabolic pathways to endure low levels of oxygen. In this section, we will describe the role of nitrate reductase (NR) and fumarate reductase (FRD) as a component of an alternative respiratory chain utilized by mycobacteria for survival during hypoxia and anoxia. It must be noted that these modulations only help mycobacteria to survive but do not facilitate mycobacterial growth.

### Nitrate Reductase

**Figure 6:**
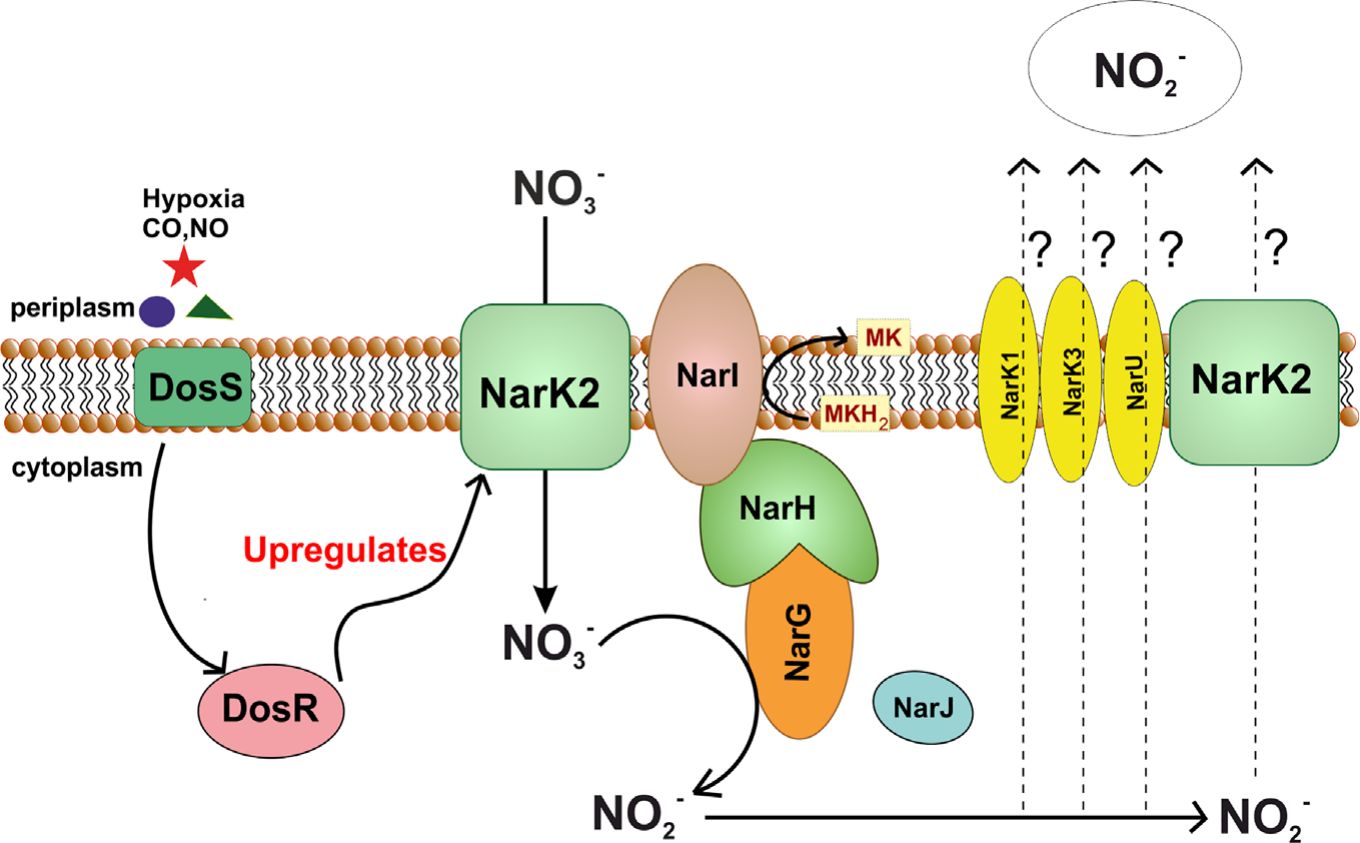
Nitrate reductase pathway in *M.* *tuberculosis*. Figure shows dependence of nitrate reductase pathway upon DosS-DosR two component system of Mtb. In stress conditions such as hypoxia, DosR upregulates expression of NarK2, which in turn, transports nitrate into the cytoplasm. This nitrate is reduced to nitrite with the help of NarGHI. In this process, electron from menaquinol are used for reduction of nitrate. Nitrate produced in the process is transported out, however the nitrate transporter remains to be identified.

NRs are families of enzymes that catalyze the reduction of nitrate ${\text{(NO}}_{3}^{-})$ into nitrite ${\text{(NO}}_{2}^{-})$. The presence of this family of enzymes in bacteria was revealed way back in 1925 through the demonstration of nitrate aided anoxic bacterial growth (Quastel et al., 1925; Stewart, 1988). These enzymes are utilized for a multitude of functions ranging from respiration to assimilation and dissimilation of nitrate. A common defining characteristic of these enzymes is the utilization of the molybdenum cofactor for the reduction of nitrate (Morozkina and Zvyagilskaya, 2007). The catalytic molybdenum site is coordinated through bis-molybdopterin guanine dinucleotide (Diaes et al., 1999). Three types of NRs are found in bacteria, i.e., cytoplasmic assimilatory, membrane-bound respiratory, and periplasmic dissimilatory (Morozkina and Zvyagilskaya, 2007). Both the periplasmic dissimilatory and membrane-bound respiratory forms are known to assist growth during anaerobic conditions and are present in *E. coli* (Stewart et al., 2002; Morozkina and Zvyagilskaya, 2007). For this review, we will primarily focus on the membrane-anchored respiratory NR as it is the only functional NR in mycobacteria (**Figure 6**). Respiratory NR is composed of three subunits (Stewart, 1988; Morozkina and Zvyagilskaya, 2007). Subunit γ is encoded by gene *narI* and it anchors the enzymatic complex to the membrane and captures electrons from reduced menaquinone using heme cofactors. Electrons are then passed onto the subunit β encoded by *narH*. NarH contains [Fe-S] clusters that facilitate the transfer of electrons to subunit α encoded by *narG*. Subunit α utilizes [Fe-S] and molybdenum cofactor to reduce nitrate into nitrite in the cytoplasm (Stewart, 1988; Morozkina and Zvyagilskaya, 2007). The expression of *narGHIJ* operon encoding the enzyme complex is tightly regulated by transcription factors like FNR, NarL, and NarP during anaerobic conditions or in the presence of nitrate (Stewart, 1994).

Mtb possesses the capability to reduce nitrate into nitrite while other related mycobacterial species lack this ability. Significantly, this ability is historically utilized for the discrimination of Mtb from *M. bovis* (Virtanen, 1960; Escoto and de Kantor, 1978). Mtb has two genetic clusters bearing homology to genes encoding dissimilatory NRs, namely *narGHJI* operon and the *narX* gene of the *narK2X* operon (Sohaskey and Wayne, 2003). The *narGHJI* operon encodes for the classical respiratory NR. Overexpression of this operon manifests NR activity in *M. smegmatis* (Weber et al., 2000), and can complement the *nar* mutant of *E. coli* for NR activity (Sohaskey and Wayne, 2003). On the other hand, NarX is a fused NR that seems to lack functional NR activity since the deletion of this gene does not affect the NR activity of Mtb (Sohaskey and Wayne, 2003). In comparison, the disruption of NarG leads to the attenuation of NR activity in Mtb. Interestingly, the aerobic cultures of Mtb possess basal levels of NR activity, which is enhanced during adaptation to hypoxia (Wayne and Hayes, 1998). However, unlike the *E. coli* *narGHJI* operon, that is induced upon hypoxia and in the presence of nitrate through transcriptional regulators FNR and NarL, the expression of *narGHJI* operon of Mtb is not induced in response to hypoxia or nitrate (Sohaskey and Wayne, 2003). On the contrary, the increase in NR activity of Mtb in response hypoxia is associated with increased expression of nitrate transporter NarK2X (Sohaskey and Wayne, 2003). Mtb possesses three isomers of nitrate transporter NarK2X, namely NarK1, NarK3, and NarU. Out of these, NarK2X is part of the dos/dormancy regulon regulated by the DosRST two-component system that responds to hypoxia, carbon monoxide and nitric oxide (Park et al., 2003; Voskuil et al., 2003; Kumar et al., 2007; Kumar et al., 2008; Trivedi et al., 2012). In line with this, the expression of *narK2* is induced during infection in mice (Shi et al., 2005b), suggesting that NR could aid in the survival of Mtb during hypoxia. However, the *narG* mutant is not attenuated for survival in hypoxia in the presence or absence of nitrate in the media (Sohaskey and Wayne, 2003). Furthermore, the expression of the *narK2* and *NarX* are downregulated upon biofilm formation (Trivedi et al., 2016). These observations raise doubts on the role of the *narGHJI* operon (and NRs) in mycobacterial survival and respiration under anaerobic conditions. However, NR protects the hypoxic Mtb cells from the stress of reactive nitrogen species and acidic pH (Tan et al., 2010).

Mtb is known for its capability to shift down its metabolic state and acquire the state of non-replicating persistence in response to a gradual decrease in the oxygen levels (Kumar et al., 2011; Bhat et al., 2012). However, Mtb does not survive sudden anaerobiosis; the presence of nitrate in culture medium aids in the survival of Mtb during sudden anaerobiosis. Furthermore, NarG plays an essential role in this survival against the sudden removal of oxygen (Sohaskey, 2008). In agreement with these observations, mycobacterial survival in the presence of respiratory inhibitor Thioridazine is enhanced by nitrate and a functional NR (Sohaskey, 2008). Inhibition of respiration leads to the accumulation of NADH (Bhat et al., 2016). Accumulated NADH ultimately leads to oxidative stress (Mavi et al., 2020), and thus it is prudent for mycobacterial cells to modulate its respiration and metabolism to re-oxidize NADH.

An interesting property of NR of Mtb is that it is not oxygen sensitive, unlike other bacterial NRs (Pichinoty and D’Ornano, 1961). Mtb utilizes this property of NR and leads to NarG dependent accumulation of nitrite in Mtb infected human macrophages (Cunningham-Bussel et al., 2013). Nitrite affects the metabolism and growth of Mtb (Cunningham-Bussel et al., 2013). It was suggested that NR aid in the maintenance of redox homeostasis in Mtb (Sohaskey, 2008). Importantly, transcripts of NarG were detected in the pericavity and the distant lung regions in clinical lung samples (Rachman et al., 2006). Furthermore, the evidence for the importance of nitrate respiration in TB pathogenesis comes from the vaccine strain *M. bovis* BCG. Although BCG exhibit the low activity of NR, however, this activity is important for survival and tissue-specific persistence of BCG in immunocompetent and immunocompromised mice (Fritz et al., 2002). Despite the importance of NR in TB pathogenesis, it has not been used as a target for drug development. Although, a few compounds like Pentachlorophenol are known to inhibit the NR. These are used primarily as a pesticide and a disinfectant. However, the establishment of NR as a valid drug target and development of Mtb specific inhibitors need further research.

### Fumarate Reductase and Bidirectional Succinate Dehydrogenases

**Figure 7:**
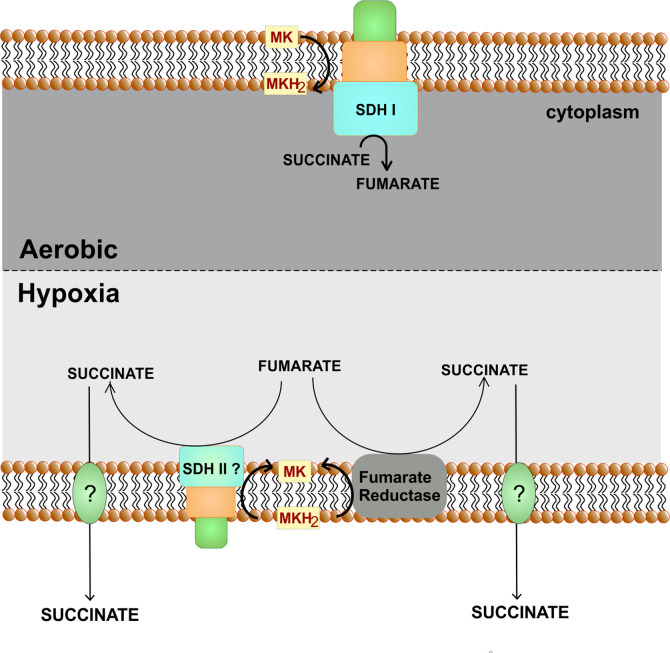
Role of fumarate reductase/succinate dehydrogenase as an alternate respiratory pathway. Under hypoxic conditions, fumarate can act as an alternate electron acceptor. For this either fumarate reductase is utilized or succinate dehydrogenase perform the reverse reaction. Succinate is secreted out of the cells.

Fumarate reductases (FRD) are the enzymes that catalyze the reduction of fumarate into succinate (Van Hellemond and Tielens, 1994). These enzymes are critical for organisms utilizing fumarate as the terminal electron acceptor. These enzymes are very similar to succinate dehydrogenases that catalyze the reverse reaction and convert succinate into fumarate while donating electrons to ubiquinone/menaquinone. Mtb genome encodes for a fumarate dehydrogenase (FRD, encoded by *frdA-rv1556* operon) and two succinate dehydrogenases (SDH1, encoded by *rv0247c–rv0249c* and SDH2, encoded by *rv3316–rv3319*) (Cook et al., 2014; Iqbal et al., 2018). FRD can be found as a soluble enzyme, but the one that participates in the respiration is often membrane-anchored (Van Hellemond and Tielens, 1994). It is a polypeptide complex consisting of three to four subunits named; A, B, C, and D. Catalytic core is constituted by FRD-A and FRD-B, whereas other domain/s act as membrane anchor and harvest electrons from menaquinone/ubiquinone (Van Hellemond and Tielens, 1994). Importantly, the FRD of Mtb is induced upon exposure to hypoxia (Watanabe et al., 2011). Furthermore, hypoxic Mtb cultures accumulate succinate in media (Watanabe et al., 2011; Eoh and Rhee, 2013). Notably, during hypoxia, Mtb could utilize a reductive half TCA cycle to oxidize NADH (Eoh and Rhee, 2013). These observations suggest that FRD will be critical for the survival of Mtb during hypoxia and in animal models. However, the *frdA* deletion mutant is not attenuated for survival during hypoxia or inside animals (Watanabe et al., 2011). Furthermore, cultures of *frdA* deletion mutant in hypoxia lead to the accumulation of equivalent levels of succinate in the media, as is observed with wild type control. These observations point towards redundancy in the FRD and SDH function in Mtb. In line with these, *sdh2* of *M. smegmatis* is upregulated during hypoxia while *sdh1* is induced during energy limiting conditions (Berney and Cook, 2010). These observations again suggest a functional redundancy (**Figure 7**). However, analysis of Mtb SDH1 and SDH 2 suggested a subtle difference in the two. It was observed that the deletion of SDH1 results in an alteration in the rate of respiration associated with the inability to recover from the stationary phase (Hartman et al., 2014). Nonetheless, such a functional redundancy is a major roadblock in targeting FRD/SDH of Mtb and suggests that inhibitors capable of inhibiting all three enzymes despite genetics and structural differences may target FRD/SDH of Mtb (Hards et al., 2020).

## Synergy Between Inhibitors of Terminal Respiratory Oxidases

Emerging literature suggests that Mtb shows a high level of flexibility in utilizing both of its terminal respiratory oxidases for survival and/or growth (Arora et al., 2014; Kalia et al., 2017b; Moosa et al., 2017; Scherr et al., 2018; Beites et al., 2019). Knock out strains of Mtb lacking *bc_1_-aa_3_* oxidoreductase (Beites et al., 2019) or cytochrome *bd* oxidase (Moosa et al., 2017) have been created, suggesting these oxidases could suffice each other’s function and thus are redundant. These observations raise questions over the suitability of the terminal oxidases as a drug target. Notably, mutants lacking a functional cytochrome *bd* oxidase are hyper susceptible to the inhibitors of *bc_1_-aa_3_* oxidoreductase (Arora et al., 2014; Kalia et al., 2017a; Moosa et al., 2017), and mutants lacking functional *bc_1_-aa_3_* oxidoreductase are highly susceptible to inhibitors of *bd* oxidase (Lu et al., 2015). These studies suggest that simultaneous inhibition of both terminal respiratory oxidases is bactericidal *in vitro* and *in vivo* (Kalia et al., 2017a; Beites et al., 2019). Importantly, such an approach is successful at killing replicating as well as non-replicating persistent bacteria. We believe that the synergistic inhibition of both the terminal oxidases represents an ideal drug development strategy (**Figure 5**). It was recently demonstrated that *bc_1_-aa_3_* oxidoreductase inhibitor Q203 and ATP synthase inhibitor leads to increased respiration in Mtb cells (Lamprecht et al., 2016). Importantly, this study also elucidated that a combination of BDQ, Q203, and clofazimine is extremely potent at killing mycobacterial cells in culture medium (Lamprecht et al., 2016). Testing animal infection with such a regiment and its comparison vis a vis isoniazid/rifampicin/pyrazinamide/ethambutol could further establish the validity of bioenergetics as one of the best drug targets available. It is important to note that BDQ lack bactericidal activity during the initial 3-4 days although its exposure leads to depletion of ATP within 3 h (Koul et al., 2014). It could be hypothesized that Mtb may survive during first few days in the presence of BDQ through limited substrate-level phosphorylation. Glycolysis is a major catabolic pathway that leads to production of ATP in Mtb cells. In the light of these, we propose that the inclusion of a glycolysis inhibitor such as 2-deoxyglucose (Jin et al., 2016) may synergize with the combination of BDQ, Q203, and clofazimine and thus could be very effective at killing mycobacterial cells *in vitro* and *in vivo*. Such a hypothesis needs to be tested to increase the efficacy of respiratory inhibitors in the mycobacterial killing. Furthermore, transposon mutagenesis based screens could be utilized for discovering pathways utilized by Mtb for generation of ATP in the presence of BDQ. Development of inhibitors of these pathways could be prioritized. Once such inhibitors are identified, they could be used in combination with BDQ and clofazimine.

## Concluding Remarks

Mycobacterial ETC is highly flexible and efficiently synchronizes with the plasticity of the central metabolic system to facilitate the survival of Mtb in varied microenvironments ranging from diverse types of granulomas to sputum droplets. During the last few years, inhibitors of ETC have raced ahead in the clinical trials, and few are even approved for the treatment of MDR-TB and XDR-TB. Amongst the components of ETC, terminal oxidases stand out as a druggable target. Several classes of chemical compounds capable of inhibiting cytochrome *bc_1_-aa_3_* oxidase are known, including Imidazopyridine amides, Arylvinylpiperazine amide, Morpholinothiophene, Pyrrolopyridinedione, Quinazolinamines, Quinolinones, Benzimidazoles, Pyrazolopyridine amides, Imidazothiazole amide, Imidazopyridine ethers, 2-(Quinolin-4-yloxy)acetamides. However, only a few classes of chemical compounds capable of inhibiting the cytochrome *bd* oxidase are known, including quinone analogs of the Aurachin family, a quinolone derivative CK-2-63, and a novel cytochrome *bd* oxidase specific inhibitor, MQL-H2, of imidazole class. Evidence emerging from hyper-susceptibility of one terminal oxidase mutant to the inhibitors of other terminal oxidase opens up the door for developing a new regimen where a combination of inhibitors targeting both the terminal oxidases can be used for the treatment of TB. In this direction, identifying novel cytochrome *bd* oxidase inhibitors should be a priority. Combination therapy could help in the shortening of the treatment duration. A regimen targeting several different pathways such as oxidative phosphorylation, cell wall biosynthesis, and RNA/protein biogenesis could emerge as a universal regimen capable of treating drug-susceptible, MDR-TB, and XDR-TB patients. TB community, along with the pharma companies, have embarked upon this mission, which has led to the strengthening of the TB drug pipeline. We are optimistic that this will lead to the evolution of new regimens for TB treatment in the future.

## Author Contributions

SB and AK conceptualized the structure of the manuscript. SB, NB, MK, GP, VC, and AK wrote the manuscript. NB and SB also helped with the illustrations of the manuscript. All authors contributed to the article and approved the submitted version.

## Funding

This work is supported by Swarnajayanti Fellowship (DST/SJF/LSA-02/2016-17) and National Bioscience Award (BT/HRD-NBA-NWB/37/01/2018) from DST and DBT, Govt of India, respectively.

## Conflict of Interest

The authors declare that the research was conducted in the absence of any commercial or financial relationships that could be construed as a potential conflict of interest.

## Abbreviations

*Mycobacterium tuberculosis*; TB, Tuberculosis; WHO, The world health organization; DOTS, Directly observed therapy short-course; MDR, Multidrug-resistant; XDR, Extensively drug-resistant; ETC, Electron transport chain; ADP, Adenosine diphosphate; ATP, Adenosine triphosphate; NADH, Reduced nicotinamide adenine dinucleotide; FADH, Reduced flavin adenine dinucleotide; SDH, Succinate dehydrogenase; NTMs, Non-tuberculous mycobacteria; SAR, Structure–activity relationship; PRSAF1, Prokaryotic respiratory supercomplex association factor 1; SOD, Superoxide dismutase; ROS, Reactive oxygen species; BCG, Bacillus Calmette Guerin; IP, Imidazo[1,2-a]pyridine; IPA, Imidazopyridine amides; MIC, Minimum inhibitory concentration; PK, Pharmacokinetic; ADME, Absorption, distribution, metabolism and excretion; TCA, Tricarboxylic acid cycle; GSK, GlaxoSmithKline; QOAs, 2-(quinolin-4-4-yloxy)acetamides; PAB, Phenoxy-alkyl benzimidazole; LPZ, Lansoprazole; LPZS, Lansoprazole sulfide; ROS, Reactive oxygen species; RNA, Ribonucleic acid.

## References

[B1] Abrahams K. A. Cox J. A. Spivey V. L. Loman N. J. Pallen M. J. Constantinidou C. (2012). Identification of novel imidazo[1,2-a]pyridine inhibitors targeting M. tuberculosis QcrB. PLoS One 7, e52951. 10.1371/journal.pone.0052951 23300833 PMC3534098

[B2] Al-Attar S. Yu Y. Pinkse M. Hoeser J. Friedrich T. Bald D. (2016). Cytochrome bd Displays Significant Quinol Peroxidase Activity. Sci. Rep. 6, 27631. 10.1038/srep27631 27279363 PMC4899803

[B3] Ananthan S. Faaleolea E. R. Goldman R. C. Hobrath J. V. Kwong C. D. Laughon B. E. (2009). High-throughput screening for inhibitors of Mycobacterium tuberculosis H37Rv. Tuberc. (Edinb.) 89, 334–353. 10.1016/j.tube.2009.05.008 PMC3255569 19758845

[B4] Andries K. Verhasselt P. Guillemont J. Gohlmann H. W. Neefs J. M. Winkler H. (2005). A diarylquinoline drug active on the ATP synthase of Mycobacterium tuberculosis. Science 307, 223–227. 10.1126/science.1106753 15591164

[B5] Arora K. Ochoa-Montano B. Tsang P. S. Blundell T. L. Dawes S. S. Mizrahi V. (2014). Respiratory flexibility in response to inhibition of cytochrome C oxidase in Mycobacterium tuberculosis. Antimicrob. Agents Chemother. 58, 6962–6965. 10.1128/AAC.03486-14 25155596 PMC4249445

[B6] Aung H. L. Berney M. Cook G. M. (2014). Hypoxia-activated cytochrome bd expression in Mycobacterium smegmatis is cyclic AMP receptor protein dependent. J. Bacteriol. 196, 3091–3097. 10.1128/JB.01771-14 24936051 PMC4135660

[B7] Bald D. Villellas C. Lu P. Koul A. (2017). Targeting Energy Metabolism in Mycobacterium tuberculosis, a New Paradigm in Antimycobacterial Drug Discovery. mBio 8, 1–11. 10.1128/mBio.00272-17 PMC5388804 28400527

[B8] Ballell L. Bates R. H. Young R. J. Alvarez-Gomez D. Alvarez-Ruiz E. Barroso V. (2013). Fueling open-source drug discovery: 177 small-molecule leads against tuberculosis. ChemMedChem 8, 313–321. 10.1002/cmdc.201200428 23307663 PMC3743164

[B9] Baughn A. D. Malamy M. H. (2004). The strict anaerobe Bacteroides fragilis grows in and benefits from nanomolar concentrations of oxygen. Nature 427, 441–444. 10.1038/nature02285 14749831

[B10] Beebout C. J. Eberly A. R. Werby S. H. Reasoner S. A. Brannon J. R. De S. (2019). Respiratory Heterogeneity Shapes Biofilm Formation and Host Colonization in Uropathogenic Escherichia coli. mBio 10, 1–16. 10.1128/mBio.02400-18 PMC6445943 30940709

[B11] Beites T. O’Brien K. Tiwari D. Engelhart C. A. Walters S. Andrews J. (2019). Plasticity of the Mycobacterium tuberculosis respiratory chain and its impact on tuberculosis drug development. Nat. Commun. 10, 4970. 10.1038/s41467-019-12956-2 31672993 PMC6823465

[B12] Berney M. Cook G. M. (2010). Unique flexibility in energy metabolism allows mycobacteria to combat starvation and hypoxia. PLoS One 5, e8614. 10.1371/journal.pone.0008614 20062806 PMC2799521

[B13] Berney M. Hartman T. E. Jacobs W. R. Jr. (2014). A Mycobacterium tuberculosis cytochrome bd oxidase mutant is hypersensitive to bedaquiline. mBio 5, e01275–e01214. 10.1128/mBio.01275-14 25028424 PMC4161257

[B14] Berry E. A. Guergova-Kuras M. Huang L. S. Crofts A. R. (2000). Structure and function of cytochrome bc complexes. Annu. Rev. Biochem. 69, 1005–1075. 10.1146/annurev.biochem.69.1.1005 10966481

[B15] Bhat S. A. Singh N. Trivedi A. Kansal P. Gupta P. Kumar A. (2012). The mechanism of redox sensing in Mycobacterium tuberculosis. Free Radic. Biol. Med. 53, 1625–1641. 10.1016/j.freeradbiomed.2012.08.008 22921590

[B16] Bhat S. A. Iqbal I. K. Kumar A. (2016). Imaging the NADH:NAD(+) Homeostasis for Understanding the Metabolic Response of Mycobacterium to Physiologically Relevant Stresses. Front. Cell Infect. Microbiol. 6, 145. 10.3389/fcimb.2016.00145 27878107 PMC5099167

[B17] Borisov V. B. Gennis R. B. Hemp J. Verkhovsky M. I. (2011). The cytochrome bd respiratory oxygen reductases. Biochim. Biophys. Acta 1807, 1398–1413. 10.1016/j.bbabio.2011.06.016 21756872 PMC3171616

[B18] Borsoi A. F. Paz J. D. Abbadi B. L. Macchi F. S. Sperotto N. Pissinate K. (2020). Design, synthesis, and evaluation of new 2-(quinoline-4-yloxy)acetamide-based antituberculosis agents. Eur. J. Med. Chem. 192, 112179. 10.1016/j.ejmech.2020.112179 32113048

[B19] Capaldi R. A. (1990). Structure and function of cytochrome c oxidase. Annu. Rev. Biochem. 59, 569–596. 10.1146/annurev.bi.59.070190.003033 2165384

[B20] Chandrasekera N. S. Alling T. Bailey M. A. Files M. Early J. V. Ollinger J. (2015). Identification of Phenoxyalkylbenzimidazoles with Antitubercular Activity. J. Med. Chem. 58, 7273–7285. 10.1021/acs.jmedchem.5b00546 26295286

[B21] Chandrasekera N. S. Berube B. J. Shetye G. Chettiar S. O’Malley T. Manning A. (2017). Improved Phenoxyalkylbenzimidazoles with Activity against Mycobacterium tuberculosis Appear to Target QcrB. ACS Infect. Dis. 3, 898–916. 10.1021/acsinfecdis.7b00112 29035551 PMC5727484

[B22] Cheng Y. Moraski G. C. Cramer J. Miller M. J. Schorey J. S. (2014). Bactericidal activity of an imidazo[1, 2-a]pyridine using a mouse M. tuberculosis infection model. PLoS One 9, e87483. 24498115 10.1371/journal.pone.0087483 PMC3909116

[B23] Chong S. M. S. Manimekalai M. S. S. Sarathy J. P. Williams Z. C. Harold L. K. Cook G. M. (2020). Antituberculosis Activity of the Antimalaria Cytochrome bcc Oxidase Inhibitor SCR0911. ACS Infect. Dis. 6, 725–737. 10.1021/acsinfecdis.9b00408 32092260

[B24] Cleghorn L. A. T. Ray P. C. Odingo J. Kumar A. Wescott H. Korkegian A. (2018). Identification of Morpholino Thiophenes as Novel Mycobacterium tuberculosis Inhibitors, Targeting QcrB. J. Med. Chem. 61, 6592–6608. 10.1021/acs.jmedchem.8b00172 29944372 PMC6089501

[B25] Cole S. T. Eiglmeier K. Parkhill J. James K. D. Thomson N. R. Wheeler P. R. (2001). Massive gene decay in the leprosy bacillus. Nature 409, 1007–1011. 10.1038/35059006 11234002

[B26] Cook G. M. Loder C. Soballe B. Stafford G. P. Membrillo-Hernandez J. Poole R. K. (1998). A factor produced by Escherichia coli K-12 inhibits the growth of E. coli mutants defective in the cytochrome bd quinol oxidase complex: enterochelin rediscovered. Microbiology 144 (Pt 12), 3297–3308. 9884221 10.1099/00221287-144-12-3297

[B27] Cook G. M. Berney M. Gebhard S. Heinemann M. Cox R. A. Danilchanka O. (2009). Physiology of mycobacteria. Adv. Microb. Physiol. 55, 81–182, 318-9. 10.1016/S0065-2911(09)05502-7 19573696 PMC3728839

[B28] Cook G. M. Hards K. Vilcheze C. Hartman T. Berney M. (2014). Energetics of Respiration and Oxidative Phosphorylation in Mycobacteria. Microbiol. Spectr. 2, 1–20. 10.1128/9781555818845.ch20 PMC4205543 25346874

[B29] Cook G. M. Hards K. Dunn E. Heikal A. Nakatani Y. Greening C. (2017). Oxidative Phosphorylation as a Target Space for Tuberculosis: Success, Caution, and Future Directions. Microbiol. Spectr. 5. 10.1128/9781555819569.ch14 PMC5480969 28597820

[B30] Crofts A. R. Shinkarev V. P. Kolling D. R. Hong S. (2003). The modified Q-cycle explains the apparent mismatch between the kinetics of reduction of cytochromes c1 and bH in the bc1 complex. J. Biol. Chem. 278, 36191–36201. 10.1074/jbc.M305461200 12829696

[B31] Cruz-Ramos H. Cook G. M. Wu G. Cleeter M. W. Poole R. K. (2004). Membrane topology and mutational analysis of Escherichia coli CydDC, an ABC-type cysteine exporter required for cytochrome assembly. Microbiology 150, 3415–3427. 10.1099/mic.0.27191-0 15470119

[B32] Cunningham-Bussel A. Zhang T. Nathan C. F. (2013). Nitrite produced by Mycobacterium tuberculosis in human macrophages in physiologic oxygen impacts bacterial ATP consumption and gene expression. Proc. Natl. Acad. Sci. U. S. A. 110, E4256–E4265. 10.1073/pnas.1316894110 24145454 PMC3831502

[B33] da Silva P. E. Von Groll A. Martin A. Palomino J. C. (2011). Efflux as a mechanism for drug resistance in Mycobacterium tuberculosis. FEMS Immunol. Med. Microbiol. 63, 1–9. 10.1111/j.1574-695X.2011.00831.x 21668514

[B34] Debnath J. Siricilla S. Wan B. Crick D. C. Lenaerts A. J. Franzblau S. G. (2012). Discovery of selective menaquinone biosynthesis inhibitors against Mycobacterium tuberculosis. J. Med. Chem. 55, 3739–3755. 10.1021/jm201608g 22449052 PMC3375340

[B35] Diase J. M. Than M. E. Humm A. Huber R. Bourenkov G. P. Bartunik H. D. (1999). Crystal structure of the first dissimilatory nitrate reductase at 1.9 A solved by MAD methods. Structure 7, 65–79. 10.1016/S0969-2126(99)80010-0 10368307

[B36] D’Mello R. Hill S. Poole R. K. (1996). The cytochrome bd quinol oxidase in Escherichia coli has an extremely high oxygen affinity and two oxygen-binding haems: implications for regulation of activity in vivo by oxygen inhibition. Microbiology 142 (Pt 4), 755–763. 10.1099/00221287-142-4-755 8936304

[B37] Dudkina N. V. Kudryashev M. Stahlberg H. Boekema E. J. (2011). Interaction of complexes I, III, and IV within the bovine respirasome by single particle cryoelectron tomography. Proc. Natl. Acad. Sci. U. S. A. 108, 15196–15200. 10.1073/pnas.1107819108 21876144 PMC3174662

[B38] Endley S. McMurray D. Ficht T. A. (2001). Interruption of the cydB locus in Brucella abortus attenuates intracellular survival and virulence in the mouse model of infection. J. Bacteriol. 183, 2454–2462. 10.1128/JB.183.8.2454-2462.2001 11274104 PMC95161

[B39] Eoh H. Rhee K. Y. (2013). Multifunctional essentiality of succinate metabolism in adaptation to hypoxia in Mycobacterium tuberculosis. Proc. Natl. Acad. Sci. U. S. A. 110, 6554–6559. 10.1073/pnas.1219375110 23576728 PMC3631649

[B40] Escoto M. L. de Kantor I. N. (1978). Nitrate reductase activity of Mycobacterium tuberculosis and Mycobacterium bovis in the presence of electron donors. J. Clin. Microbiol. 7, 601–602. 97306 10.1128/jcm.7.6.601-602.1978 PMC275083

[B41] Foo C. S. Lupien A. Kienle M. Vocat A. Benjak A. Sommer R. (2018). Arylvinylpiperazine Amides, a New Class of Potent Inhibitors Targeting QcrB of Mycobacterium tuberculosis. mBio 9, 1–13. 10.1128/mBio.01276-18 PMC6178619 30301850

[B42] Fritz C. Maass S. Kreft A. Bange F. C. (2002). Dependence of Mycobacterium bovis BCG on anaerobic nitrate reductase for persistence is tissue specific. Infect. Immun. 70, 286–291. 10.1128/IAI.70.1.286-291.2002 11748194 PMC127612

[B43] Galvan A. E. Chalon M. C. Rios Colombo N. S. Schurig-Briccio L. A. Sosa-Padilla B. Gennis R. B. (2019). Microcin J25 inhibits ubiquinol oxidase activity of purified cytochrome bd-I from Escherichia coli. Biochimie 160, 141–147. 10.1016/j.biochi.2019.02.007 30790617

[B44] Giacobbo B. C. Pissinate K. Rodrigues-Junior V. Villela A. D. Grams E. S. Abbadi B. L. (2017). New insights into the SAR and drug combination synergy of 2-(quinolin-4-yloxy)acetamides against Mycobacterium tuberculosis. Eur. J. Med. Chem. 126, 491–501. 10.1016/j.ejmech.2016.11.048 27914363

[B45] Gong H. Li J. Xu A. Tang Y. Ji W. Gao R. (2018). An electron transfer path connects subunits of a mycobacterial respiratory supercomplex. Science 362, 1–11. 10.1126/science.aat8923 30361386

[B46] Hao W. Golding G. B. (2006). Asymmetrical evolution of cytochrome bd subunits. J. Mol. Evol. 62, 132–142. 10.1007/s00239-005-0005-7 16474982

[B47] Hards K. Adolph C. Harold L. K. McNeil M. B. Cheung C. Y. Jinich A. (2020). Two for the price of one: Attacking the energetic-metabolic hub of mycobacteria to produce new chemotherapeutic agents. Prog. Biophys. Mol. Biol. 152, 35–44. 10.1016/j.pbiomolbio.2019.11.003 31733221

[B48] Harikishore A. Chong S. S. M. Ragunathan P. Bates R. W. Gruber G. (2020). Targeting the menaquinol binding loop of mycobacterial cytochrome bd oxidase. Mol. Divers. 10.1007/s11030-020-10034-0 31939065

[B49] Harrison G. A. Mayer Bridwell A. E. Singh M. Jayaraman K. Weiss L. A. Kinsella R. L. (2019). Identification of 4-Amino-Thieno[2,3-d]Pyrimidines as QcrB Inhibitors in Mycobacterium tuberculosis. mSphere 4, 1–14. 10.1128/mSphere.00606-19 PMC6739496 31511370

[B50] Hartman T. Weinrick B. Vilcheze C. Berney M. Tufariello J. Cook G. M. (2014). Succinate dehydrogenase is the regulator of respiration in Mycobacterium tuberculosis. PLoS Pathog. 10, e1004510. 10.1371/journal.ppat.1004510 25412183 PMC4239112

[B51] Hong W. D. Gibbons P. D. Leung S. C. Amewu R. Stocks P. A. Stachulski A. (2017). Synthesis, and Biological Evaluation of Heterocyclic Quinolones Targeting the Respiratory Chain of Mycobacterium tuberculosis. J. Med. Chem. 60, 3703–3726. 10.1021/acs.jmedchem.6b01718 28304162

[B52] Hori H. Tsubaki M. Mogi T. Anraku Y. (1996). EPR study of NO complex of bd-type ubiquinol oxidase from Escherichia coli. J. Biol. Chem. 271, 9254–9258. 10.1074/jbc.271.16.9254 8621585

[B53] Iqbal I. K. Bajeli S. Akela A. K. Kumar A. (2018). Bioenergetics of Mycobacterium: An Emerging Landscape for Drug Discovery. Pathogens 7, 1–30. 10.3390/pathogens7010024 PMC5874750 29473841

[B54] Jang J. Kim R. Woo M. Jeong J. Park D. E. Kim G. (2017). Efflux Attenuates the Antibacterial Activity of Q203 in Mycobacterium tuberculosis. Antimicrob. Agents Chemother. 61, 1–8. 10.1128/AAC.02637-16 PMC5487614 28416541

[B55] Jin T. Mehrens H. Wang P. Kim S. G. (2016). Glucose metabolism-weighted imaging with chemical exchange-sensitive MRI of 2-deoxyglucose (2DG) in brain: Sensitivity and biological sources. Neuroimage 143, 82–90. 10.1016/j.neuroimage.2016.08.040 27570111 PMC5124509

[B56] Junemann S. Wrigglesworth J. M. (1995). Cytochrome bd oxidase from Azotobacter vinelandii. Purification and quantitation of ligand binding to the oxygen reduction site. J. Biol. Chem. 270, 16213–16220. 10.1074/jbc.270.27.16213 7608187

[B57] Juty N. S. Moshiri F. Merrick M. Anthony C. Hill S. (1997). The Klebsiella pneumoniae cytochrome bd’ terminal oxidase complex and its role in microaerobic nitrogen fixation. Microbiology 143 (Pt 8), 2673–2683. 10.1099/00221287-143-8-2673 9274021

[B58] Kabus A. Niebisch A. Bott M. (2007). Role of cytochrome bd oxidase from Corynebacterium glutamicum in growth and lysine production. Appl. Environ. Microbiol. 73, 861–868. 10.1128/AEM.01818-06 17142369 PMC1800781

[B59] Kalia N. P. Hasenoehrl E. J. Ab Rahman N. B. Koh V. H. Ang M. L. T. Sajorda D. R. (2017a). Exploiting the synthetic lethality between terminal respiratory oxidases to kill Mycobacterium tuberculosis and clear host infection. Proc. Natl. Acad. Sci. U. S. A. 114, 7426–7431. 10.1073/pnas.1706139114 28652330 PMC5514758

[B60] Kalia N. P. Hasenoehrl E. J. Ab Rahman N. B. Koh V. H. Ang M. L. T. Sajorda D. R. (2017b). Exploiting the synthetic lethality between terminal respiratory oxidases to kill Mycobacterium tuberculosis and clear host infection. Proc. Natl. Acad. Sci. U. S. A. 114, 7426–7431. 10.1073/pnas.1706139114 28652330 PMC5514758

[B61] Kana B. D. Weinstein E. A. Avarbock D. Dawes S. S. Rubin H. Mizrahi V. (2001). Characterization of the cydAB-encoded cytochrome bd oxidase from Mycobacterium smegmatis. J. Bacteriol. 183, 7076–7086. 10.1128/JB.183.24.7076-7086.2001 11717265 PMC95555

[B62] Kang S. Kim R. Y. Seo M. J. Lee S. Kim Y. M. Seo M. (2014). Lead optimization of a novel series of imidazo[1,2-a]pyridine amides leading to a clinical candidate (Q203) as a multi- and extensively-drug-resistant anti-tuberculosis agent. J. Med. Chem. 57, 5293–5305. 10.1021/jm5003606 24870926

[B63] Kao W. C. Kleinschroth T. Nitschke W. Baymann F. Neehaul Y. Hellwig P. (2016). The obligate respiratory supercomplex from Actinobacteria. Biochim. Biophys. Acta 1857, 1705–1714. 10.1016/j.bbabio.2016.07.009 27472998

[B64] Kashket E. R. (1985). The proton motive force in bacteria: a critical assessment of methods. Annu. Rev. Microbiol. 39, 219–242. 10.1146/annurev.mi.39.100185.001251 2998266

[B65] Keam S. J. (2019). Pretomanid: First Approval. Drugs 79, 1797–1803. 10.1007/s40265-019-01207-9 31583606

[B66] Kim M. S. Jang J. Ab Rahman N. B. Pethe K. Berry E. A. Huang L. S. (2015). Isolation and Characterization of a Hybrid Respiratory Supercomplex Consisting of Mycobacterium tuberculosis Cytochrome bcc and Mycobacterium smegmatis Cytochrome aa3. J. Biol. Chem. 290, 14350–14360. 10.1074/jbc.M114.624312 25861988 PMC4505504

[B67] Ko Y. Choi I. (2016). Putative 3D Structure of QcrB from Mycobacterium tuberculosis Cytochrome bc1 Complex, a Novel Drug-Target for New Series of Antituberculosis Agent Q203. Bull. Korean Chem. Soc. 37, 725–731. 10.1002/bkcs.10765

[B68] Koul A. Vranckx L. Dhar N. Gohlmann H. W. Ozdemir E. Neefs J. M. (2014). Delayed bactericidal response of Mycobacterium tuberculosis to bedaquiline involves remodelling of bacterial metabolism. Nat. Commun. 5, 3369. 10.1038/ncomms4369 24569628 PMC3948051

[B69] Krause F. Reifschneider N. H. Vocke D. Seelert H. Rexroth S. Dencher N. A. (2004). “Respirasome”-like supercomplexes in green leaf mitochondria of spinach. J. Biol. Chem. 279, 48369–48375. 10.1074/jbc.M406085200 15342644

[B70] Kucera I. Hedbavny R. Dadak V. (1988). Separate binding sites for antimycin and mucidin in the respiratory chain of the bacterium Paracoccus denitrificans and their occurrence in other denitrificans bacteria. Biochem. J. 252, 905–908. 10.1042/bj2520905 2844159 PMC1149234

[B71] Kumar A. Toledo J. C. Patel R. P. Lancaster J. R. Jr. Steyn A. J. (2007). Mycobacterium tuberculosis DosS is a redox sensor and DosT is a hypoxia sensor. Proc. Natl. Acad. Sci. U. S. A. 104, 11568–11573. 10.1073/pnas.0705054104 17609369 PMC1906723

[B72] Kumar A. Deshane J. S. Crossman D. K. Bolisetty S. Yan B. S. Kramnik I. (2008). Heme oxygenase-1-derived carbon monoxide induces the Mycobacterium tuberculosis dormancy regulon. J. Biol. Chem. 283, 18032–18039. 10.1074/jbc.M802274200 18400743 PMC2440631

[B73] Kumar A. Farhana A. Guidry L. Saini V. Hondalus M. Steyn A. J. (2011). Redox homeostasis in mycobacteria: the key to tuberculosis control? Expert Rev. Mol. Med. 13, e39. 22172201 10.1017/S1462399411002079 PMC3241215

[B74] Kusumoto K. Sakiyama M. Sakamoto J. Noguchi S. Sone N. (2000). Menaquinol oxidase activity and primary structure of cytochrome bd from the amino-acid fermenting bacterium Corynebacterium glutamicum. Arch. Microbiol. 173, 390–397. 10.1007/s002030000161 10896219

[B75] Lamprecht D. A. Finin P. M. Rahman M. A. Cumming B. M. Russell S. L. Jonnala S. R. (2016). Turning the respiratory flexibility of Mycobacterium tuberculosis against itself. Nat. Commun. 7, 12393. 10.1038/ncomms12393 27506290 PMC4987515

[B76] Larsen M. H. Kallipolitis B. H. Christiansen J. K. Olsen J. E. Ingmer H. (2006). The response regulator ResD modulates virulence gene expression in response to carbohydrates in Listeria monocytogenes. Mol. Microbiol. 61, 1622–1635. 10.1111/j.1365-2958.2006.05328.x 16968229

[B77] Lee B. S. Sviriaeva E. Pethe K. (2020). Targeting the cytochrome oxidases for drug development in mycobacteria. Prog. Biophys. Mol. Biol. 152, 45–54. 10.1016/j.pbiomolbio.2020.02.001 32081616

[B78] Lenaz G. Genova M. L. (2010). Structure and organization of mitochondrial respiratory complexes: a new understanding of an old subject. Antioxid. Redox Signal 12, 961–1008. 10.1089/ars.2009.2704 19739941

[B79] Li X. W. Herrmann J. Zang Y. Grellier P. Prado S. Muller R. (2013). Synthesis and biological activities of the respiratory chain inhibitor aurachin D and new ring versus chain analogues. Beilstein J. Org. Chem. 9, 1551–1558. 10.3762/bjoc.9.176 23946854 PMC3740496

[B80] Lindqvist A. Membrillo-Hernandez J. Poole R. K. Cook G. M. (2000). Roles of respiratory oxidases in protecting Escherichia coli K12 from oxidative stress. Antonie Van Leeuwenhoek 78, 23–31. 10.1023/A:1002779201379 11016692

[B81] Lu P. Heineke M. H. Koul A. Andries K. Cook G. M. Lill H. (2015). The cytochrome bd-type quinol oxidase is important for survival of Mycobacterium smegmatis under peroxide and antibiotic-induced stress. Sci. Rep. 5, 10333. 10.1038/srep10333 26015371 PMC4450806

[B82] Lu P. Asseri A. H. Kremer M. Maaskant J. Ummels R. Lill H. (2018). The anti-mycobacterial activity of the cytochrome bcc inhibitor Q203 can be enhanced by small-molecule inhibition of cytochrome bd. Sci. Rep. 8, 2625. 10.1038/s41598-018-20989-8 29422632 PMC5805707

[B83] Lu X. Williams Z. Hards K. Tang J. Cheung C. Y. Aung H. L. (2019). Pyrazolo[1,5-a]pyridine Inhibitor of the Respiratory Cytochrome bcc Complex for the Treatment of Drug-Resistant Tuberculosis. ACS Infect. Dis. 5, 239–249. 10.1021/acsinfecdis.8b00225 30485737

[B84] Lupien A. Foo C. S. Savina S. Vocat A. Piton J. Monakhova N. (2020). New 2-Ethylthio-4-methylaminoquinazoline derivatives inhibiting two subunits of cytochrome bc1 in Mycobacterium tuberculosis. PLoS Pathog. 16, e1008270. 10.1371/journal.ppat.1008270 31971990 PMC6999911

[B85] Lv K. Li L. Wang B. Liu M. Shen W. Guo H. (2017). Design, synthesis and antimycobacterial activity of novel imidazo[1,2-a]pyridine-3-carboxamide derivatives. Eur. J. Med. Chem. 137, 117–125. 10.1016/j.ejmech.2017.05.044 28577507

[B86] Magalon A. Alberge F. (2016). Distribution and dynamics of OXPHOS complexes in the bacterial cytoplasmic membrane. Biochim. Biophys. Acta 1857, 198–213. 10.1016/j.bbabio.2015.10.015 26545610

[B87] Malpica R. Franco B. Rodriguez C. Kwon O. Georgellis D. (2004). Identification of a quinone-sensitive redox switch in the ArcB sensor kinase. Proc. Natl. Acad. Sci. U. S. A. 101, 13318–13323. 10.1073/pnas.0403064101 15326287 PMC516565

[B88] Manjunatha U. Boshoff H. I. Barry C. E. (2009). The mechanism of action of PA-824: Novel insights from transcriptional profiling. Commun. Integr. Biol. 2, 215–218. 10.4161/cib.2.3.7926 19641733 PMC2717523

[B89] Mascolo L. Bald D. (2020). Cytochrome bd in Mycobacterium tuberculosis: A respiratory chain protein involved in the defense against antibacterials. Prog. Biophys. Mol. Biol. 152, 55–63. 10.1016/j.pbiomolbio.2019.11.002 31738981

[B90] Matsoso L. G. Kana B. D. Crellin P. K. Lea-Smith D. J. Pelosi A. Powell D. (2005). Function of the cytochrome bc1-aa3 branch of the respiratory network in mycobacteria and network adaptation occurring in response to its disruption. J. Bacteriol. 187, 6300–6308. 10.1128/JB.187.18.6300-6308.2005 16159762 PMC1236647

[B91] Mavi P. S. Singh S. Kumar A. (2020). Reductive Stress: New Insights in Physiology and Drug Tolerance of Mycobacterium. Antioxid. Redox Signal 32, 1348–1366. 10.1089/ars.2019.7867 31621379

[B92] Mdanda S. Baijnath S. Shobo A. Singh S. D. Maguire G. E. M. Kruger H. G. (2017). Lansoprazole-sulfide, pharmacokinetics of this promising anti-tuberculous agent. BioMed. Chromatogr. 31. 10.1002/bmc.4035 28623874

[B93] Megehee J. A. Lundrigan M. D. (2007). Temporal expression of Mycobacterium smegmatis respiratory terminal oxidases. Can. J. Microbiol. 53, 459–463. 10.1139/W06-140 17538658

[B94] Megehee J. A. Hosler J. P. Lundrigan M. D. (2006). Evidence for a cytochrome bcc-aa3 interaction in the respiratory chain of Mycobacterium smegmatis. Microbiology 152, 823–829. 10.1099/mic.0.28723-0 16514162

[B95] Meunier B. Madgwick S. A. Reil E. Oettmeier W. Rich P. R. (1995). New inhibitors of the quinol oxidation sites of bacterial cytochromes bo and bd. Biochemistry 34, 1076–1083. 10.1021/bi00003a044 7827023

[B96] Miller M. J. Gennis R. B. (1983). The purification and characterization of the cytochrome d terminal oxidase complex of the Escherichia coli aerobic respiratory chain. J. Biol. Chem. 258, 9159–9165. 6307994

[B97] Mitchell P. (1976). Possible molecular mechanisms of the protonmotive function of cytochrome systems. J. Theor. Biol. 62, 327–367. 10.1016/0022-5193(76)90124-7 186667

[B98] Mogi T. Ui H. Shiomi K. Omura S. Kita K. (2008). Gramicidin S identified as a potent inhibitor for cytochrome bd-type quinol oxidase. FEBS Lett. 582, 2299–2302. 10.1016/j.febslet.2008.05.031 18519036

[B99] Mogi T. Ui H. Shiomi K. Omura S. Miyoshi H. Kita K. (2009). Antibiotics LL-Z1272 identified as novel inhibitors discriminating bacterial and mitochondrial quinol oxidases. Biochim. Biophys. Acta 1787, 129–133. 10.1016/j.bbabio.2008.11.016 19111521

[B100] Moosa A. Lamprecht D. A. Arora K. Barry C. E. Boshoff H. I. M. Ioerger T. R. (2017). Susceptibility of Mycobacterium tuberculosis Cytochrome bd Oxidase Mutants to Compounds Targeting the Terminal Respiratory Oxidase, Cytochrome c. Antimicrob. Agents Chemother. 61, 1–5. 10.1128/AAC.01338-17 PMC5610507 28760899

[B101] Moraski G. C. Markley L. D. Cramer J. Hipskind P. A. Boshoff H. Bailey M. (2013). Advancement of Imidazo[1,2-a]pyridines with Improved Pharmacokinetics and Nanomolar Activity Against Mycobacterium tuberculosis. ACS Med. Chem. Lett. 4, 675–679. 10.1021/ml400088y 23930153 PMC3733398

[B102] Moraski G. C. Miller P. A. Bailey M. A. Ollinger J. Parish T. Boshoff H. I. (2015). Putting Tuberculosis (TB) To Rest: Transformation of the Sleep Aid, Ambien, and “Anagrams” Generated Potent Antituberculosis Agents. ACS Infect. Dis. 1, 85–90. 10.1021/id500008t 25984566 PMC4426345

[B103] Moraski G. C. Cheng Y. Cho S. Cramer J. W. Godfrey A. Masquelin T. (2016a). Imidazo[1,2-a]Pyridine-3-Carboxamides Are Active Antimicrobial Agents against Mycobacterium avium Infection In Vivo. Antimicrob. Agents Chemother. 60, 5018–5022. 10.1128/AAC.00618-16 27216051 PMC4958206

[B104] Moraski G. C. Seeger N. Miller P. A. Oliver A. G. Boshoff H. I. Cho S. (2016b). Arrival of Imidazo[2,1-b]thiazole-5-carboxamides: Potent Anti-tuberculosis Agents That Target QcrB. ACS Infect. Dis. 2, 393–398. 10.1021/acsinfecdis.5b00154 27627627

[B105] Moraski G. C. Deboosere N. Marshall K. L. Weaver H. A. Vandeputte A. Hastings C. (2020). Intracellular and in vivo evaluation of imidazo[2,1-b]thiazole-5-carboxamide anti-tuberculosis compounds. PLoS One 15, e0227224. 10.1371/journal.pone.0227224 31905374 PMC6944458

[B106] Morozkina E. V. Zvyagilskaya R. A. (2007). Nitrate reductases: structure, functions, and effect of stress factors. Biochem. (Mosc.) 72, 1151–1160. 10.1134/S0006297907100124 18021072

[B107] Mulkidjanian A. Y. (2007). Proton translocation by the cytochrome bc1 complexes of phototrophic bacteria: introducing the activated Q-cycle. Photochem. Photobiol. Sci. 6, 19–34. 10.1039/B517522D 17200733

[B108] Niebisch A. Bott M. (2001). Molecular analysis of the cytochrome bc1-aa3 branch of the Corynebacterium glutamicum respiratory chain containing an unusual diheme cytochrome c1. Arch. Microbiol. 175, 282–294. 10.1007/s002030100262 11382224

[B109] Niebisch A. Bott M. (2003). Purification of a cytochrome bc-aa3 supercomplex with quinol oxidase activity from Corynebacterium glutamicum. Identification of a fourth subunity of cytochrome aa3 oxidase and mutational analysis of diheme cytochrome c1. J. Biol. Chem. 278, 4339–4346. 10.1074/jbc.M210499200 12446663

[B110] Pan Z. Wang Y. Gu X. Wang J. Cheng M. (2019). Refined homology model of cytochrome bcc complex B subunit for virtual screening of potential anti-tuberculosis agents. J. Biomol. Struct. Dyn. 38, 1–13. 10.1080/07391102.2019.1688196 31674290

[B111] Parish T. Smith D. A. Roberts G. Betts J. Stoker N. G. (2003). The senX3-regX3 two-component regulatory system of Mycobacterium tuberculosis is required for virulence. Microbiology 149, 1423–1435. 10.1099/mic.0.26245-0 12777483

[B112] Park H. D. Guinn K. M. Harrell M. I. Liao R. Voskuil M. I. Tompa M. (2003). Rv3133c/dosR is a transcription factor that mediates the hypoxic response of Mycobacterium tuberculosis. Mol. Microbiol. 48, 833–843. 10.1046/j.1365-2958.2003.03474.x 12694625 PMC1992516

[B113] Pecsi I. Hards K. Ekanayaka N. Berney M. Hartman T. Jacobs W. R. Jr. (2014). Essentiality of succinate dehydrogenase in Mycobacterium smegmatis and its role in the generation of the membrane potential under hypoxia. mBio 5, 1–14. 10.1128/mBio.01093-14 PMC4145680 25118234

[B114] Pethe K. Bifani P. Jang J. Kang S. Park S. Ahn S. (2013). Discovery of Q203, a potent clinical candidate for the treatment of tuberculosis. Nat. Med. 19, 1157–1160. 10.1038/nm.3262 23913123

[B115] Phummarin N. Boshoff H. I. Tsang P. S. Dalton J. Wiles S. Barry Rd C. E. (2016). SAR and identification of 2-(quinolin-4-yloxy)acetamides as Mycobacterium tuberculosis cytochrome bc1 inhibitors. Medchemcomm 7, 2122–2127. 10.1039/C6MD00236F 28337336 PMC5292992

[B116] Pichinoty F. D’Ornano L. (1961). Inhibition by oxygen of biosynthesis and activity of nitrate-reductase in Aerobacter aerogenes. Nature 191, 879–881. 10.1038/191879a0 13735447

[B117] Pissinate K. Villela A. D. Rodrigues-Junior V. Giacobbo B. C. Grams E. S. Abbadi B. L. (2016). 2-(Quinolin-4-yloxy)acetamides Are Active against Drug-Susceptible and Drug-Resistant Mycobacterium tuberculosis Strains. ACS Med. Chem. Lett. 7, 235–239. 10.1021/acsmedchemlett.5b00324 26985307 PMC4789679

[B118] Pitta E. Rogacki M. K. Balabon O. Huss S. Cunningham F. Lopez-Roman E. M. (2016). Searching for New Leads for Tuberculosis: Design, Synthesis, and Biological Evaluation of Novel 2-Quinolin-4-yloxyacetamides. J. Med. Chem. 59, 6709–6728. 10.1021/acs.jmedchem.6b00245 27348630

[B119] Poole R. K. Cook G. M. (2000). Redundancy of aerobic respiratory chains in bacteria? Routes, reasons and regulation. Adv. Microb. Physiol. 43, 165–224. 10.1016/S0065-2911(00)43005-5 10907557

[B120] Quastel J. H. Stephenson M. Whetham M. D. (1925). Some Reactions of Resting Bacteria in Relation to Anaerobic Growth. Biochem. J. 19, 304–317. 10.1042/bj0190304 16743505 PMC1259178

[B121] Rachman H. Strong M. Ulrichs T. Grode L. Schuchhardt J. Mollenkopf H. (2006). Unique transcriptome signature of Mycobacterium tuberculosis in pulmonary tuberculosis. Infect. Immun. 74, 1233–1242. 10.1128/IAI.74.2.1233-1242.2006 16428773 PMC1360294

[B122] Rao S. P. Alonso S. Rand L. Dick T. Pethe K. (2008). The protonmotive force is required for maintaining ATP homeostasis and viability of hypoxic, nonreplicating Mycobacterium tuberculosis. Proc. Natl. Acad. Sci. U. S. A. 105, 11945–11950. 10.1073/pnas.0711697105 18697942 PMC2575262

[B123] Roberts G. Vadrevu I. S. Madiraju M. V. Parish T. (2011). Control of CydB and GltA1 expression by the SenX3 RegX3 two component regulatory system of Mycobacterium tuberculosis. PLoS One 6, e21090. 10.1371/journal.pone.0021090 21698211 PMC3116866

[B124] Ryan N. J. Lo J. H. (2014). Delamanid: first global approval. Drugs 74, 1041–1045. 10.1007/s40265-014-0241-5 24923253

[B125] Rybniker J. Vocat A. Sala C. Busso P. Pojer F. Benjak A. (2015). Lansoprazole is an antituberculous prodrug targeting cytochrome bc1. Nat. Commun. 6, 7659. 10.1038/ncomms8659 26158909 PMC4510652

[B126] Safarian S. Rajendran C. Muller H. Preu J. Langer J. D. Ovchinnikov S. (2016). Structure of a bd oxidase indicates similar mechanisms for membrane-integrated oxygen reductases. Science 352, 583–586. 10.1126/science.aaf2477 27126043 PMC5515584

[B127] Safarian S. Hahn A. Mills D. J. Radloff M. Eisinger M. L. Nikolaev A. (2019). Active site rearrangement and structural divergence in prokaryotic respiratory oxidases. Science 366, 100–104. 10.1126/science.aay0967 31604309

[B128] Sakamoto J. Matsumoto A. Oobuchi K. Sone N. (1996). Cytochrome bd-type quinol oxidase in a mutant of Bacillus stearothermophilus deficient in caa3-type cytochrome c oxidase. FEMS Microbiol. Lett. 143, 151–158. 10.1111/j.1574-6968.1996.tb08474.x 8837467

[B129] Scherr N. Bieri R. Thomas S. S. Chauffour A. Kalia N. P. Schneide P. (2018). Targeting the Mycobacterium ulcerans cytochrome bc1:aa3 for the treatment of Buruli ulcer. Nat. Commun. 9, 5370. 10.1038/s41467-018-07804-8 30560872 PMC6299076

[B130] Scott R. A. (1995). Functional significance of cytochrome c oxidase structure. Structure 3, 981–986. 10.1016/S0969-2126(01)00233-7 8590007

[B131] Seung K. J. Keshavjee S. Rich M. L. (2015). Multidrug-Resistant Tuberculosis and Extensively Drug-Resistant Tuberculosis. Cold Spring Harb. Perspect. Med. 5, a017863. 10.1101/cshperspect.a017863 25918181 PMC4561400

[B132] Shi L. Sohaskey C. D. Kana B. D. Dawes S. North R. J. Mizrahi V. (2005a). Changes in energy metabolism of Mycobacterium tuberculosis in mouse lung and under in vitro conditions affecting aerobic respiration. Proc. Natl. Acad. Sci. U. S. A. 102, 15629–15634. 10.1073/pnas.0507850102 16227431 PMC1255738

[B133] Shi L. Sohaskey C. D. Kana B. D. Dawes S. North R. J. Mizrahi V. (2005b). Changes in energy metabolism of Mycobacterium tuberculosis in mouse lung and under in vitro conditions affecting aerobic respiration. Proc. Natl. Acad. Sci. U. S. A. 102, 15629–15634. 10.1073/pnas.0507850102 16227431 PMC1255738

[B134] Singh N. Kumar A. (2015). Virulence factor SenX3 is the oxygen-controlled replication switch of Mycobacterium tuberculosis. Antioxid. Redox Signal 22, 603–613. 10.1089/ars.2014.6020 25333974 PMC4333250

[B135] Small J. L. Park S. W. Kana B. D. Ioerger T. R. Sacchettini J. C. Ehrt S. (2013). Perturbation of cytochrome c maturation reveals adaptability of the respiratory chain in Mycobacterium tuberculosis. mBio 4, e00475–e00413. 10.1128/mBio.00475-13 24045640 PMC3781833

[B136] Sohaskey C. D. Wayne L. G. (2003). Role of narK2X and narGHJI in hypoxic upregulation of nitrate reduction by Mycobacterium tuberculosis. J. Bacteriol. 185, 7247–7256. 10.1128/JB.185.24.7247-7256.2003 14645286 PMC296237

[B137] Sohaskey C. D. (2008). Nitrate enhances the survival of Mycobacterium tuberculosis during inhibition of respiration. J. Bacteriol. 190, 2981–2986. 10.1128/JB.01857-07 18296525 PMC2293237

[B138] Sone N. Fukuda M. Katayama S. Jyoudai A. Syugyou M. Noguchi S. (2003). QcrCAB operon of a nocardia-form actinomycete Rhodococcus rhodochrous encodes cytochrome reductase complex with diheme cytochrome cc subunit. Biochim. Biophys. Acta 1557, 125–131. 10.1016/S0005-2728(02)00394-8 12615356

[B139] Stewart V. Lu Y. Darwin A. J. (2002). Periplasmic nitrate reductase (NapABC enzyme) supports anaerobic respiration by Escherichia coli K-12. J. Bacteriol. 184, 1314–1323. 10.1128/JB.184.5.1314-1323.2002 11844760 PMC134854

[B140] Stewart V. (1988). Nitrate respiration in relation to facultative metabolism in enterobacteria. Microbiol. Rev. 52, 190–232. 10.1128/MMBR.52.2.190-232.1988 3045516 PMC373136

[B141] Stewart V. (1994). Regulation of nitrate and nitrite reductase synthesis in enterobacteria. Antonie Van Leeuwenhoek 66, 37–45. 10.1007/BF00871631 7747939

[B142] Sviriaeva E. Subramanian Manimekalai M. S. Gruber G. Pethe K. (2020). Features and Functional Importance of Key Residues of the Mycobacterium tuberculosis Cytochrome bd Oxidase. ACS Infect. Dis. 6, 1697–1707. 10.1021/acsinfecdis.9b00449 32379966

[B143] Tan M. P. Sequeira P. Lin W. W. Phong W. Y. Cliff P. Ng S. H. (2010). Nitrate respiration protects hypoxic Mycobacterium tuberculosis against acid- and reactive nitrogen species stresses. PLoS One 5, e13356. 10.1371/journal.pone.0013356 21048946 PMC2965054

[B144] Tantry S. J. Markad S. D. Shinde V. Bhat J. Balakrishnan G. Gupta A. K. (2017). Discovery of Imidazo[1,2-a]pyridine Ethers and Squaramides as Selective and Potent Inhibitors of Mycobacterial Adenosine Triphosphate (ATP) Synthesis. J. Med. Chem. 60, 1379–1399. 10.1021/acs.jmedchem.6b01358 28075132

[B145] Thakare R. Soni I. Dasgupta A. Chopra S. (2015). Delamanid for the treatment of pulmonary multidrug-resistant tuberculosis. Drugs Today (Barc.) 51, 117–123. 10.1358/dot.2015.51.2.2245645 25756067

[B146] Theßeling A. Rasmussen T. Burschel S. Wohlwend D. Kagi J. Muller R. (2019). Homologous bd oxidases share the same architecture but differ in mechanism. Nat. Commun. 10, 5138. 10.1038/s41467-019-13122-4 31723136 PMC6853902

[B147] Tran S. L. Cook G. M. (2005). The F1Fo-ATP synthase of Mycobacterium smegmatis is essential for growth. J. Bacteriol. 187, 5023–5028. 10.1128/JB.187.14.5023-5028.2005 15995221 PMC1169501

[B148] Trivedi A. Singh N. Bhat S. A. Gupta P. Kumar A. (2012). Redox biology of tuberculosis pathogenesis. Adv. Microb Physiol. 60, 263–324. 10.1016/B978-0-12-398264-3.00004-8 22633061

[B149] Trivedi A. Mavi P. S. Bhatt D. Kumar A. (2016). Thiol reductive stress induces cellulose-anchored biofilm formation in Mycobacterium tuberculosis. Nat. Commun. 7, 11392. 10.1038/ncomms11392 27109928 PMC4848537

[B150] Turner A. K. Barber L. Z. Wigley P. Muhammad S. Jones M. A. Lovell M. A. (2003). Contribution of proton-translocating proteins to the virulence of Salmonella enterica serovars Typhimurium, Gallinarum, and Dublin in chickens and mice. Infect. Immun. 71, 3392–3401. 10.1128/IAI.71.6.3392-3401.2003 12761123 PMC155768

[B151] van der Westhuyzen R. Winks S. Wilson C. R. Boyle G. A. Gessner R. K. Soares de Melo C. (2015). Pyrrolo[3,4-c]pyridine-1,3(2H)-diones: A Novel Antimycobacterial Class Targeting Mycobacterial Respiration. J. Med. Chem. 58, 9371–9381. 10.1021/acs.jmedchem.5b01542 26551248

[B152] Van Hellemond J. J. Tielens A. G. (1994). Expression and functional properties of fumarate reductase. Biochem. J. 304 ( Pt 2), 321–331. 10.1042/bj3040321 7998964 PMC1137495

[B153] Virtanen S. (1960). A study of nitrate reduction by mycobacteria. The use of the nitrate reduction test in the identification of mycobacteria. Acta Tuberc. Scand. Suppl 48, 1–119. 13842446

[B154] Voggu L. Schlag S. Biswas R. Rosenstein R. Rausch C. Gotz F. (2006). Microevolution of cytochrome bd oxidase in Staphylococci and its implication in resistance to respiratory toxins released by Pseudomonas. J. Bacteriol 188, 8079–8086. 10.1128/JB.00858-06 17108291 PMC1698191

[B155] von Jagow G. Link T. A. (1986). Use of specific inhibitors on the mitochondrial bc1 complex. Methods Enzymol. 126, 253–271. 10.1016/S0076-6879(86)26026-7 2856132

[B156] von Jagow G. Ljungdahl P. O. Graf P. Ohnishi T. Trumpower B. L. (1984). An inhibitor of mitochondrial respiration which binds to cytochrome b and displaces quinone from the iron-sulfur protein of the cytochrome bc1 complex. J. Biol. Chem. 259, 6318–6326. 6327677

[B157] Von Jagow G. Gribble G. W. Trumpower B. L. (1986). Mucidin and strobilurin A are identical and inhibit electron transfer in the cytochrome bc1 complex of the mitochondrial respiratory chain at the same site as myxothiazol. Biochemistry 25, 775–780. 10.1021/bi00352a006 3008811

[B158] Voskuil M. I. Schnappinger D. Visconti K. C. Harrell M. I. Dolganov G. M. Sherman D. R. (2003). Inhibition of respiration by nitric oxide induces a Mycobacterium tuberculosis dormancy program. J. Exp. Med. 198, 705–713. 10.1084/jem.20030205 12953092 PMC2194188

[B159] W.H. Organisation (2010). Treatment of tuberculosis: guidelines. (Geneva: World Health Organization). 23741786

[B160] W.H. Organisation (2019). Global Tuberculosis Report 2019. (Geneva: World Health Organization), 283.

[B161] W.H. Organization (2013). The Use of Bedaquiline in the Treatment of Multidrug-Resistant Tuberculosis: Interim Policy Guidance, The Use of Bedaquiline in the Treatment of Multidrug-Resistant Tuberculosis: Interim Policy Guidance (Geneva: World Health Organization). 26110189

[B162] Walker J. E. (2013). The ATP synthase: the understood, the uncertain and the unknown. Biochem. Soc. Trans. 41, 1–16. 10.1042/BST20110773 23356252

[B163] Ward S. A. Biagini G. A. Nixon G. L. O’Neill P. M. (2017). “Combination product,” in WIPO (United Kingdom: PCT WIPO, Liverpool School Of Tropical Medicine), 102.

[B164] Watanabe S. Zimmermann M. Goodwin M. B. Sauer U. Barry C. E. Boshoff H. I. (2011). Fumarate reductase activity maintains an energized membrane in anaerobic Mycobacterium tuberculosis. PLoS Pathog. 7, e1002287. 10.1371/journal.ppat.1002287 21998585 PMC3188519

[B165] Wayne L. G. Hayes L. G. (1998). Nitrate reduction as a marker for hypoxic shiftdown of Mycobacterium tuberculosis. Tuberc. Lung Dis. 79, 127–132. 10.1054/tuld.1998.0015 10645451

[B166] Weber I. Fritz C. Ruttkowski S. Kreft A. Bange F. C. (2000). Anaerobic nitrate reductase (narGHJI) activity of Mycobacterium bovis BCG in vitro and its contribution to virulence in immunodeficient mice. Mol. Microbiol. 35, 1017–1025. 10.1046/j.1365-2958.2000.01794.x 10712684

[B167] Winstedt L. Yoshida K. Fujita Y. von Wachenfeldt C. (1998). Cytochrome bd biosynthesis in Bacillus subtilis: characterization of the cydABCD operon. J. Bacteriol. 180, 6571–6580. 10.1128/JB.180.24.6571-6580.1998 9852001 PMC107760

[B168] Wiseman B. Nitharwal R. G. Fedotovskaya O. Schafer J. Guo H. Kuang Q. (2018). Structure of a functional obligate complex III2IV2 respiratory supercomplex from Mycobacterium smegmatis. Nat. Struct. Mol. Biol. 25, 1128–1136. 10.1038/s41594-018-0160-3 30518849

[B169] Wu Z. Lu Y. Li L. Zhao R. Wang B. Lv K. (2016). Identification of N-(2-Phenoxyethyl)imidazo[1,2-a]pyridine-3-carboxamides as New Antituberculosis Agents. ACS Med. Chem. Lett. 7, 1130–1133. 10.1021/acsmedchemlett.6b00330 27994751 PMC5150680

[B170] Yamamoto Y. Poyart C. Trieu-Cuot P. Lamberet G. Gruss A. Gaudu P. (2005). Respiration metabolism of Group B Streptococcus is activated by environmental haem and quinone and contributes to virulence. Mol. Microbiol. 56, 525–534. 10.1111/j.1365-2958.2005.04555.x 15813741

[B171] Zhang-Barber L. Turner A. K. Martin G. Frankel G. Dougan G. Barrow P. A. (1997). Influence of genes encoding proton-translocating enzymes on suppression of Salmonella typhimurium growth and colonization. J. Bacteriol. 179, 7186–7190. 10.1128/JB.179.22.7186-7190.1997 9371470 PMC179664

